# Identification and Analysis of SARS-CoV-2 Alpha Variants in the Largest Taiwan COVID-19 Outbreak in 2021

**DOI:** 10.3389/fmed.2022.869818

**Published:** 2022-04-25

**Authors:** Li-Teh Liu, Jih-Jin Tsai, Ko Chang, Chun-Hong Chen, Ping-Chang Lin, Ching-Yi Tsai, Yan-Yi Tsai, Miao-Chen Hsu, Wan-Long Chuang, Jer-Ming Chang, Shang-Jyh Hwang, Inn-Wen Chong

**Affiliations:** ^1^Department of Medical Laboratory Science and Biotechnology, College of Medical Technology, Chung Hwa University of Medical Technology, Tainan, Taiwan; ^2^Tropical Medicine Center, Kaohsiung Medical University Hospital, Kaohsiung, Taiwan; ^3^Division of Infectious Diseases, Department of Internal Medicine, Kaohsiung Medical University Hospital, Kaohsiung, Taiwan; ^4^School of Medicine, College of Medicine, Kaohsiung Medical University, Kaohsiung, Taiwan; ^5^Department of Internal Medicine, Kaohsiung Municipal Siaogang Hospital, Kaohsiung Medical University, Kaohsiung, Taiwan; ^6^National Mosquito-Borne Diseases Control Research Center, National Health Research Institutes, Zhunan, Taiwan; ^7^National Institute of Infectious Diseases and Vaccinology, National Health Research Institutes, Zhunan, Taiwan; ^8^Division of Hepatobiliary and Pancreatic, Kaohsiung Medical University Hospital, Kaohsiung, Taiwan; ^9^Division of Nephrology, Department of Internal Medicine, Kaohsiung Medical University Hospital, Kaohsiung, Taiwan; ^10^Department of Medical Research, Kaohsiung Medical University Hospital, Kaohsiung, Taiwan; ^11^Department of Internal Medicine and Graduate Institute of Medicine, Kaohsiung Medical University, Kaohsiung, Taiwan; ^12^Department of Pulmonary Medicine, Kaohsiung Medical University Hospital, Kaohsiung, Taiwan

**Keywords:** COVID-19, SARS-CoV-2, qRT-PCR, virus culture, next-generation sequencing, clade replacements, phylogenetic analysis, alpha/B.1.1.7

## Abstract

Severe acute respiratory syndrome coronavirus 2 (SARS-CoV-2) is believed to have originated in Wuhan City, Hubei Province, China, in December 2019. Infection with this highly dangerous human-infecting coronavirus *via* inhalation of respiratory droplets from SARS-CoV-2 carriers results in coronavirus disease 2019 (COVID-19), which features clinical symptoms such as fever, dry cough, shortness of breath, and life-threatening pneumonia. Several COVID-19 waves arose in Taiwan from January 2020 to March 2021, with the largest outbreak ever having a high case fatality rate (CFR) (5.95%) between May and June 2021. In this study, we identified five 20I (alpha, V1)/B.1.1.7/GR SARS-CoV-2 (KMUH-3 to 7) lineage viruses from COVID-19 patients in this largest COVID-19 outbreak. Sequence placement analysis using the existing SARS-CoV-2 phylogenetic tree revealed that KMUH-3 originated from Japan and that KMUH-4 to KMUH-7 possibly originated *via* local transmission. Spike mutations M1237I and D614G were identified in KMUH-4 to KMUH-7 as well as in 43 other alpha/B.1.1.7 sequences of 48 alpha/B.1.1.7 sequences deposited in GISAID derived from clinical samples collected in Taiwan between 20 April and July. However, M1237I mutation was not observed in the other 12 alpha/B.1.1.7 sequences collected between 26 December 2020, and 12 April 2021. We conclude that the largest COVID-19 outbreak in Taiwan between May and June 2021 was initially caused by the alpha/B.1.1.7 variant harboring spike D614G + M1237I mutations, which was introduced to Taiwan by China Airlines cargo crew members. To our knowledge, this is the first documented COVID-19 outbreak caused by alpha/B.1.1.7 variant harboring spike M1237I mutation thus far. The largest COVID-19 outbreak in Taiwan resulted in 13,795 cases and 820 deaths, with a high CFR, at 5.95%, accounting for 80.90% of all cases and 96.47% of all deaths during the first 2 years. The high CFR caused by SARS-CoV-2 alpha variants in Taiwan can be attributable to comorbidities and low herd immunity. We also suggest that timely SARS-CoV-2 isolation and/or sequencing are of importance in real-time epidemiological investigations and in epidemic prevention. The impact of D614G + M1237I mutations in the spike gene on the SARS-CoV-2 virus spreading as well as on high CFR remains to be elucidated.

## Introduction

Severe acute respiratory syndrome coronavirus 2 (SARS-CoV-2), previously known as 2019-nCoV (1), belongs to the *Betacoronavirus* genus, Coronaviridae family, and Nidovirales order (2). SARS-CoV-2 is the seventh coronavirus (CoV), and one of the most dangerous CoVs (3, 4), that infects humans (3–7) and can cause life-threatening coronavirus disease 2019 (COVID-19) (1). SARS-CoV-2 is a round, positive-sense, single-stranded, enveloped RNA virus with a linear genome of ∼30,000 nucleotides. Its genome is composed of 11 protein-coding sequences that encode 12 protein products (8). Phylogenetic analysis suggests that SARS-CoV-2 originated from bat SARS-like betacoronaviruses. However, its genetic and biological features are more similar to those of SARS-CoV-1 (9). This virus was isolated from human airway epithelial cells in bronchoalveolar lavage fluid samples from patients with pneumonia in Wuhan City, Hubei Province, China, on 21 December 2019 (7). Since then, SARS-CoV-2 has been detected in a wide spectrum of clinical specimens, including nasopharyngeal swabs, sputum, blood, urine, and feces (10, 11). Entry of SARS-CoV-2 into host cells is primarily mediated by the binding of its spike protein to angiotensin-converting enzyme 2 (ACE2) and other cellular factors, such as TMPRSS2 (12) and NRP1 (13). Inhalation of respiratory droplets from SARS-CoV-2 carriers during close contact, e.g., coughing, sneezing, or talking, as well as contact with virus-containing nasal or oral secretions, can result in clinical symptoms such as fever, dry cough, and shortness of breath as well as life-threatening pneumonia (14, 15). In addition, wastewater transmission pathways have been discovered, such as human infection *via* environmental sewage (16). During the COVID-19 pandemic, accumulating genetic variations have led to SARS-CoV-2 variants, which are designated variants under monitoring (VUM), variants of interest (VOI), and variants of concern (VOC). It is suggested that evolution of this virus has resulted in increasing disease severity, mortality, and transmissibility and in the development of resistance to antivirals, vaccination, and immune responses (17).

Several COVID-19 waves arose in Taiwan from January 2020 to March 2021 (18–22). In Taiwan, the first wave began from a confirmed COVID-19 case involving a 55-year-old businesswoman who returned from Wuhan City, Hubei Province, China, during the Lunar New Year holidays (24–29 January) on 21 January 2020 (23), a month after SARS-CoV-2 was isolated and identified (7). In 2019, Taiwan’s population was approximately 23.6 million, with approximately 1 million living long-term in China and approximately 400,000 working in China (24). It is estimated that approximately 40,000 people returned to Taiwan from China to celebrate the Lunar New Year holidays every year during that time (25). Since then, Taiwan has implemented and maintained stringent intervention measures, such as boarder control, contact tracing, real-time diagnosis, safe social distancing, mask wearing, frequent hand washing, and timely clinical triage of critically ill patients with appropriate medical measures (26). As of the end of December 2020, Taiwan had recorded only 873 cases and 7 deaths and was able to avoid a national lockdown (27). However, an alpha variant (B.1.1.7) COVID-19 outbreak occurred between May and June 2021. Cases in this largest COVID-19 outbreak ever in Taiwan were characterized by rapid progression from infection to death (28, 29).

In this study, we isolated SARS-CoV-2 virus from clinical samples collected from COVID-19 patients during the largest COVID-19 outbreak ever in Taiwan and performed next generation sequencing to reveal the virus strain(s) in these infections. We also analyzed SARS-CoV-2 sequences deposited in GISAID EpiCoV^1^ which were derived from clinical samples collected in Taiwan between January 2020 and December 2021 to display SARS-CoV-2 clade replacements in Taiwan in the first 2 years of the pandemic. In reviewing the special events of COVID-19 in Taiwan between 2020 and 2021, we raised the question why alpha/B.1.1.7 variant imported into Taiwan in January 2021 [special events from Daily COVID-19 Press Release by the Central Epidemic Command Center (CECC)^2^ ] did not cause a COVID-19 outbreak similar to that occurring between May and June 2021. This question unexpectedly led us to reveal the origin of this largest outbreak.

## Materials and Methods

### Ethics Statement and Sample Collection

This study was approved by the Institutional Review Board of Kaohsiung Medical University Hospital (KMUH), Kaohsiung City, Taiwan (approval no. KMUHIRB-E-I-20200013). As an authorized hospital by the CECC, Taiwan, we performed viral diagnosis for suspected COVID-19 patients. All qRT-PCR and virus culture experiments were conducted in the Tropical Medicine Center (TMC) with a biosafety level 3 laboratory and complied with the laboratory biosafety guidelines established by the Taiwan Centers for Disease Control (TCDC) (30). The nasopharyngeal swabs of suspected COVID-19 volunteers were collected in Universal Transport Medium (UTM) (Viral Transport Medium w/Special Swab, Creative Life Science, Taiwan) in KMUH. The swab-UTMs were then immediately subjected to SARS-CoV-2 qRT-PCR. The swab-UTM sample with a positive PCR result was sent for SARS-CoV-2 culture.

### RNA Extraction and SARS-CoV-2 qRT-PCR

Severe Acute Respiratory Syndrome Coronavirus 2 genomic RNA was detected by qRT-PCR using SARS-CoV-2-specific primers and probes for the E, N, and RdRP genes (31). In brief, total RNA was extracted from 140 μL swab-UTM using the QIAamp Virus RNA mini kit (QIAGEN, Germany) following the manufacturer’s instructions. One-step qRT-PCR was performed in a 20-μL mixture containing 5 μL of extracted RNA with an Mx3000P PCR System (Agilent, United States) and a LightCycler Multiplex RNA Virus Master kit (Roche Diagnostics, Germany). A cycle threshold (Ct) value <40 indicates a positive result (32). Negative (RNAse-free water) and positive (RNA extracted from hCoV-19/Taiwan/4/2020, EPI_ISL_411927 virus culture fluid) controls were included. The primers, probe, mixture, machine, and thermal cycling conditions are listed in Supplementary Tables 1A–C.

### SARS-CoV-2 Isolation Using VERO E6 Cell Culture

VERO E6 cells were used for SARS-CoV-2 propagation and routinely maintained in Dulbecco’s modified Eagle’s medium (Thermo Fisher Scientific, United States) supplemented with 10% fetal bovine serum, 1× antibiotic-antimycotic (Thermo Fisher Scientific, United States) and 1 mM sodium pyruvate (Thermo Fisher Scientific, United States) at 37°C in the presence of 5% CO_2_. The cells were seeded at a density of 1 × 10^5^ cells per well in a 24-well plate overnight for sample inoculation. One hundred microliters of swab-UTM sample with positive qRT-PCR results was inoculated into the cells in duplicate, and the cells were incubated at 37°C in the presence of 5% CO_2_ for 1 h for virus attachment. Six hundred microliters of DMEM with 2% FBS was added to the well; after incubation, the cytopathic effect (CPE) was examined daily under a phase-contrast microscope. Once the CPE was observed, the presence of SARS-CoV-2 in the culture supernatant was confirmed by qRT-PCR. For samples that did not show CPE after three days of incubation, blind passage was performed until day 21 to increase the chance for virus propagation and isolation, with medium renewal every 2–3 days.

### RNA Library Construction, Next-Generation Sequencing, and *in silico* Sequence Analysis

The RNA library for next-generation sequencing (NGS) was constructed with VAHTS Universal V8 RNA-seq Library Prep Kit (Vazyme Biotech, China) by using a total of 10^9^ copies of SARS-CoV-2 RNA; the copy number was predetermined with a COVID-19 Multiplex 1-Step RTqPCR Kit (Topgen Biotech, Taiwan). In brief, the RNA was pretreated with divalent cations at 94°C for 8 min to obtain small RNA pieces with a length of 150–200 nucleotides. Next, the small pieces of RNA were reverse-transcribed following the manufacturer’s procedures to construct a paired-end cDNA library with an average insert size of approximately 150 bp. The cDNAs were ligated to barcode sequencing adapters, and the quality of the cDNA library was analyzed using a MultiNA MCE-202 (Shimadzu, Japan) with a DNA 2500 Kit (Shimadzu, Japan). NGS of the paired-end cDNA library was performed using a NovaSeq 6000 Sequencing System (Illumina, United States) following the manufacturer’s standard protocol. Approximately twenty million paired-end reads (∼150 bp per read) were produced per cDNA library using a paired-end RNA-seq approach (Illumina, United States). The adapter sequences were trimmed from the sequence reads and filtered by using fastp (v 0.19.5) (33) with a quality value (QV) ≥ 20; the read lengths were filtered by Filter FASTQ (v1.1.5) (34) with a cut-off ≥145 bp.

### Targeted Sequencing (Multiplexed PCR) of Low-Viral Load Samples

Three sets of primer pools were used for NGS targeted whole-genome amplification of SARS-CoV-2, including 98 pairs of ARTIC V3 primers (amplicon size: 375-419 bp)^3^ from ARTIC Network^4^ and 98 pairs of custom-designed primers (amplicon size: 139–206 bp) (Supplementary Table 1D) covering the gaps of the ARTIC V3 set when sequenced with NovaSeq 6000 (Illumina, United States) PE150 reads. Briefly, cDNA was synthesized from 2 μL of extracted viral RNA using HiScript II Q RT SuperMix for qPCR (Vazyme Biotech, China) with random hexamers. Three separate PCRs were performed using AmpSeq Multi-PCR Module V2 (Vazyme Biotech, China) with three primer pools: Pool 1 contained odd-numbered ATRIC primers; Pool 2 contained even-numbered ATRIC primers; and Pool 3 contained all custom-designed primers. The PCR mixture was incubated for 2 min at 99°C for denaturation, followed by 32 cycles of 99°C for 15 s and 60°C for 4 min; an Applied Biosystems 9700 Thermal Cycler (Applied Biosystems, United States) was used. The amplified products were purified with DNA Clean Beads (Vazyme Biotech, China) to exclude small non-specific fragments. End preparation (5′phosphorylation and 3′adenine addition) was performed at 20°C for 15 min and 65°C for 15 min using a VAHTS Universal DNA Library Prep Kit V3 (Vazyme Biotech, China). Next, adapter ligation was processed with dual-barcode adapters from Illumina (Topgen Biotech, Taiwan) by 20°C for 15 min. The thermal cycling for amplification of the library was as follows: 95°C for 3 min, 20 cycles of 98 °C for 20 s, 60°C for 15 s, and 72°C for 30 s and a final extension step 72°C for 5 min. The amplified products were purified with DNA Clean Beads (Vazyme Biotech, China) to exclude non-specific fragments. The specialized amplicon sizes of Pool 1 and Pool 2 were approximately 500–550 bp, and that of Pool 3 was approximately 250–350 bp. The qualified library was further analyzed with a MultiNA MCE-202 and a DNA 2500 Kit (Shimadzu, Japan), and we performed paired-end sequencing using a NovaSeq 6000 (Illumina, United States) following the manufacturer’s recommended protocol.

### Read Mapping for Single-Nucleotide Variation/Insertion and Deletion and *de novo* Assembly

Retained reads were aligned to the reference sequence Wuhan-Hu-1/2019 (MN908947) using Burrows–Wheeler Aligner (v0.7.17.2) (35). Insertion and deletion (InDel) events were evaluated by using Dindel (v1.01) (36); single-nucleotide variation (SNV) was assessed by using Lofreq (v2.1.5) (37). Qualified sequencing reads were further manipulated using fastq-join (Version 1.1.2) (38), and all reads were assembled into contigs using Unicycler (v0.4.8.0) (39).

### Validation of the Low-Depth (<10 Reads) Region by PCR Amplification and Sanger Sequencing

RNA was reverse-transcribed by using 4× VirDect 1-step RT-qPCR Master Mix with random primers (Topgen Biotech., Taiwan) to generate cDNA. To enable a fast-sequencing approach, amplifications were performed using 10 ng cDNA with the TopPLUS PCR Master Mix (Topgen Biotech., Taiwan) and specific target primer pairs with a working concentration of 250 nM and an Applied Biosystems 9700 Thermal Cycler (Applied Biosystems, United States) according to the manufacturer’s instructions. The thermal cycling program was as follows: 95°C for 3 min, 32 cycles of 95°C for 15 s, 60°C for 20 s, and 72°C for 40 s, and a final extension at 72°C for 2 min. The amplified products were purified with VAHTS DNA Clean Beads (Vazyme Biotech., China), analyzed using a MultiNA MCE 202 with DNA 2500 Kit (Shimadzu, Japan) to check the target amplicon length and quantity. Sanger sequencing was then performed according to the manufacturer’s protocol to confirm variants and indel regions.

### SARS-CoV-2 Genomes and Evolutionary Analysis

Genomic sequences of SARS-CoV-2 derived from clinical samples collected in Taiwan between January 2020 and December 2021 were retrieved and downloaded from GISAID EpiCoV. Before reconstruction of the phylogenetic tree, the SARS-CoV-2 sequences were aligned by using MAFFT v 7.490^5^ (40), and the most appropriate evolutionary model used in the construction of the phylogenetic tree was evaluated by using ModelFinder (41). Theoretical phylogenetic trees were reconstructed with 1,000 bootstrap replicates by using IQ-TREE 2.1.3 COVID-edition (42).

### Statistical Analysis

Statistical analysis was performed using the SPSS Statistics software version 19.0 (IBM Corp., United States). The significance of the difference between groups was calculated by Chi-squared test.

## Results

### A Brief History of COVID-19 in Taiwan

**FIGURE 1:**
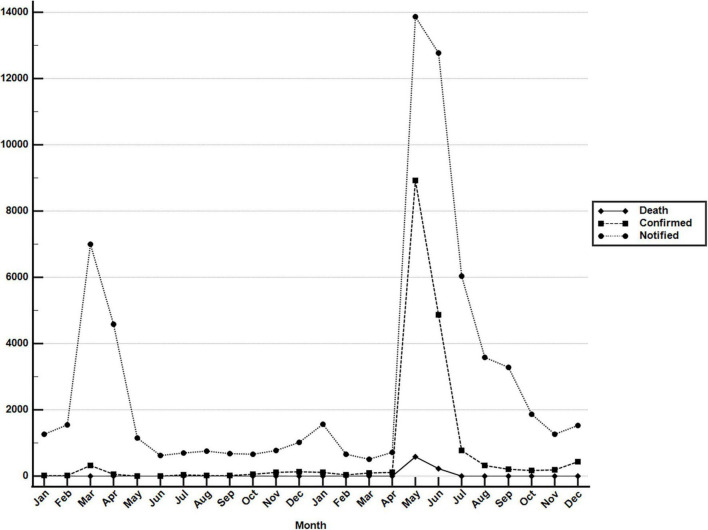
Monthly COVID-19 data between January 2020 and December 2021 in Taiwan. Monthly data of confirmed COVID-19 cases and deaths resulting from COVID-19 between 1 January 2020 and 31 December 2021. These data were retrieved from the notifiable diseases surveillance system maintained by the Taiwan CDC. Source of data: https://nidss.cdc.gov.tw/nndss/disease?id=19CoV.

**FIGURE 2:**
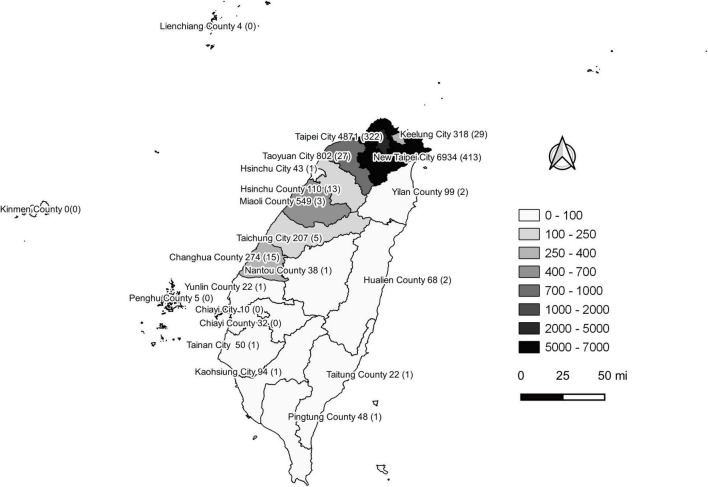
Geographical distribution of autochthonous COVID-19 cases between January 2020 and December 2021 in Taiwan. Confirmed COVID-19 case numbers are shown in each city of the second-level administrative division in Taiwan. The numbers in the brackets are fatal cases. This figure was generated using QGIS v3.16.14 (QGIS Development Team, 2022, QGIS Geographic Information System, Open Source Geospatial Foundation, http://www.qgis.org/). Taiwan map data were retrieved from the Taiwan Geospatial One-Stop Portal developed by the Information Center of the Taiwan Ministry of The Interior and used under the Open Government Data License.

The COVID-19 outbreak in Taiwan began in late January 2020, when Taiwanese businessmen, tourists and students returned to Taiwan from Wuhan City and Hubei Province, China, for the Lunar New Year holidays (14–29 January) (23). The second wave started in March, when Taiwanese businessmen, travelers and students returned to Taiwan from all over the world since the global COVID-19 pandemic began. Most COVID-19 cases identified during this period of time can be traced to where the country from which travelers returned (43). The efforts by the Taiwanese government and citizens stopped the outbreak from May to November, and the first domestic case of COVID-19 was reported in December, 253 days after the last confirmed case in April (44, 45). As of the end of 31 December 2020, Taiwan, a country of approximately 23.6 million people, had recorded only 823 cases and 9 deaths (Figure 1). During the COVID-19 pandemic, maintaining normal operations of private organizations and government departments has posed a great challenge globally. With almost no community-transmitted cases and without any complete lockdown in 2020, Taiwan is one of very few countries worldwide that has recorded a minimal impact due to the pandemic (27). The third wave occurred during the 2021 Lunar New Year holidays (11--16 February). The fourth wave in Taiwan, the largest yet, began in April and ended in September. Since then, cases have only occurred sporadically through the end of 2021. There were 17,050 confirmed COVID-19 cases between January 2020 and December 2021; 85.6% (14,600 cases) were autochthonous cases, and most were attributable to the outbreak from May to June 2021. Notably, from May to June, the chief virus strain in Taiwan was the alpha variant, which resulted in a case fatality rate (CFR) up to 5.95% (820/13,795),^6^ even higher than the global CFR (2.15%).^7^ The geographical distribution of autochthonous cases between January 2020 and December 2021 is shown in Figure 2. Most of the cases were distributed in northern and northwestern cities in Taiwan, where the population density is relatively high and traffic volume is relatively large.^8^

### Detection and Isolation of SARS-CoV-2 During the Largest COVID-19 Outbreak

Compared with most countries in the world, Taiwan has had a relatively low number of COVID-19 cases (Supplementary Figure 1). KMUH is located in Kaohsiung city in southern Taiwan, an industrial city adjacent to the Taiwan Strait, where most epidemic cases are dengue fever (46); COVID-19 cases are relatively rare in Taiwan. In the local area, patients with symptoms suspicious of COVID-19 and a contact history or SARS-CoV-2 positivity by RT-PCR or saliva screening test during border control were assigned by the CECC to KMUH for diagnosis and treatment. During the largest COVID-19 outbreak between April and June 2021, we collected nasopharyngeal swabs from suspected COVID-19 cases with upper respiratory tract syndrome. Among 13 nasopharyngeal swab-UTM samples, genomic RNA of SARS-CoV-2 was detected by qRT-PCR in all samples. We then performed SARS-CoV-2 isolation by using the VERO E6 cell line, which was established from African green monkey kidney epithelial cells and is widely used for SARS-CoV-2 culture. CPE development was observed in cell cultures inoculated with four swab-UTM samples (sample numbers 32, 36, 38, and 41) at 5–14 days postinoculation. Ct values for qRT-PCR for the E, RdRP, and N genes were between 11.88 and 24.1, relatively lower than for samples that did not show CPE. CPE was observed in phase contrast microscopy on days 6, 11, and 14 post-inoculation in cells inoculated with original nasopharyngeal swab-UTM of sample numbers 32, 38, and 41. Interestingly, CPE was observed on days 5, 8, and 7 post-inoculation, which blind passage was performed on day 3 post-inoculation, the day no CPE was observed in cells inoculated with the original nasopharyngeal swab-UTM of sample numbers 32, 38, and 41. These results were in agreement that the performance of “blind passages” increased infectivity to optimize the detection of low titers and/or slow-growing viruses (47). However, we did not observe this effect in sample number 36. To verify whether CPE was induced by SARS-CoV-2, the culture supernatant was assessed for the presence of SARS-CoV-2 genomic RNA by using qRT-PCR, and the results suggested that the CPE observed was induced by SARS-CoV-2 (Table 1). The virus isolation rate was as low as 30.8% (4/13) in this study.

**TABLE 1:** Detection of the presence of SARS-CoV-2 using qRT-PCR and VERO E6 cell CPE.

	Swab-UTM^a^ (Ct)			CPE observed DPI^b^		Culture fluid^c^ (Ct)				
Sample number	E gene	RdRP gene	N gene	Original swab	Blind passage^d^	E gene	RdRP gene	N gene	Strain	TCID_50_*^f^*
29	35.75	33.5	31.4	Negative	Negative	ND	ND	ND	–	–
30	34.96	32.36	33.5	Negative	Negative	ND	ND	ND	–	–
31	36.32	33.5	36.5	Negative	Negative	ND	ND	ND	–	–
32	24.06	23.78	24.1	6	5	10.99	15.91	20.24	KMUH–3	10^5.6^
33	35	33.8	34	Negative	Negative	ND	ND	ND	–	–
34	35	33.65	32.8	Negative	Negative	ND	ND	ND	–	–
35	36	33.53	33.8	Negative	Negative	ND	ND	ND	–	–
36	17.31	17.52	17.88	6	8	21.46	25.79	30.67	KMUH–4	10^1.5^
37	27.3	34.1	32	Negative	Negative	ND	ND	ND	KMUH–5	–
38	16.87	17.18	15.3	11	8	9.7	15.62	18.33	KMUH–6	10^4.4^
39	26.42	25.69	ND*^e^*	Negative	Negative	ND	ND	ND	–	–
40	28.81	28.32	ND	Negative	Negative	ND	ND	ND	–	–
41	13.6	11.88	15.2	14	7	10.59	15.56	19.66	KMUH–7	10^5.2^

*^a^RNA extracted from nasopharyngeal swab-UTM.*

*^b^DPI: days postinoculation.*

*^c^RNA extracted from culture supernatant from VERO E6 cells with CPE.*

*^d^Culture medium from VERO E6 cells without CPE at 3 days postinoculation was transferred to a well with fresh confluent VERO E6 cells, and CPE was examined daily until day 21.*

*^e^ND: not determined.*

*^f^TCID_50_: median tissue culture infectious dose.*

### Next-Generation Sequencing of SARS-CoV-2 and Data Analysis *in silico*

To understand the lineage or clade identity of SARS-CoV-2 detected by qRT-PCR, RNAs were extracted from the original swab-UTM samples for NGS. RNA libraries were constructed, and NGS was performed as described in section “Materials and Methods.” As RNA-seq can only be processed successfully using samples with low Ct values (Ct < 24), such as samples 32, 36, 38, and 41, a targeted sequencing method was used for samples with high Ct values (Ct > 24). However, PCR-based targeted sequencing was only successfully applied to sample 37 because RNA degradation occurring in other clinical samples interfered with the coverage of the entire virus genome. SNVs and InDels were investigated using Wuhan-Hu-1/2019 (MN908947) as a reference sequence with Dindel (v1.01) (36) and Lofreq (v2.1.5) (37), respectively. To verify the SNV and InDel results analyzed in our genome workstation, we deposited these five genomic RNA sequences into GISAID EpiCoV, as named in the order of the above numbers KMUH-3 to KMUH-7 (Table 1), and analyzed them by using Nextclade v1.10.0^3^ (48). The KMUH-4 to KMUH-7 sequences belong to the 20I (alpha, V1)/B.1.1.7/GRY (NextStrain_clade/pangolin_lineage/GISAID_clade) lineage, and KMUH-3 is 20I (alpha, V1)/B.1.1.7/GR; these were at that time the most important VOCs determined by the European Centre for Disease Prevention and Control (ECDC) and the World Health Organization (WHO). The consensus results are shown in Supplementary Table 2. Amino acid deletion events and non-synonymous codon variations are shown in Table 2. These isolates share many spike mutations of interest, such as N501Y, D614G, and P681H, and many more deletions (e.g., NSP6 3675–3677del, spike 69–70del, and spike 144del) and codon variations. However, we did not detect any insertion or frameshift events in these five SARS-CoV-2 sequences. The results of NGS coverage and depth distribution of KMUH-3 (RNA-seq) and KMUH-5 (targeted sequencing) as well as SNVs at nt positions 14,676 (NSP12 P4804P), 23,063 (spike N501Y) and 27,513 (NS7a Y40Y) of KMUH-4, which were confirmed by Sanger sequencing, are shown in Supplementary Figure 2 to demonstrate the quality of RNA-seq and targeted sequencing. The deletions and SNVs shown in Supplementary Table 2 together resulted in coverages of 95.39–99.87% and depths between 1,000 and 200,000 in the five SARS-CoV-2 sequences when compared to the reference sequence.

**TABLE 2:** Sequence variation of KMUH-3 to KMUH-7 compared to the reference Wuhan-Hu-1/2019.

			Strain^a^				
**Variant type**	**Protein**	**aa position^b^**	**KMUH-3**	**KMUH-4**	**KMUH-5**	**KMUH-6**	**KMUH-7**
Amino acid deletion	NSP6	3675–3677	X	X	X	X	X
	Spike	69–70	X	X	X	X	X
		144	X	X	X	X	X
Codon change^c^	NSP2	F343L	X	O	O	O	O
		P732S	O	O	X	O	O
	NSP3	T1001I	X	X	X	X	X
		A1708D	X	X	X	X	X
		I2230T	X	X	X	X	X
	NSP12	P314L	X	X	X	X	X
	NSP15	T2165M	X	O	O	O	O
		P2256S	O	O	O	X	O
	Spike	N501Y	X	X	X	X	X
		A570D	X	X	X	X	X
		D614G	X	X	X	X	X
		P681H	X	X	X	X	X
		T716I	X	X	X	X	X
		S982A	X	X	X	X	X
		D1118H	X	X	X	X	X
		M1237I	O	X	X	X	X
	NS7	Q90*	O	O	O	O	X
	NS8	Q27*	X	X	O	X	X
		R52I	X	X	X	X	X
		Y73C	X	X	X	X	X
	N	D3L	X	X	X	X	X
		R203K	X	X	X	X	X
		G204R	X	X	X	X	X
		S235F	X	X	X	X	X

*^a^X: with this variation, O: without this variation.*

^b^*: Stop codon.

*^c^We display only non-synonymous codon variations in this table; refer to Supplementary Table 2 for full information.*

### Possible Origin of the Five SARS-CoV-2 Sequences Identified in This Study

**FIGURE 3:**
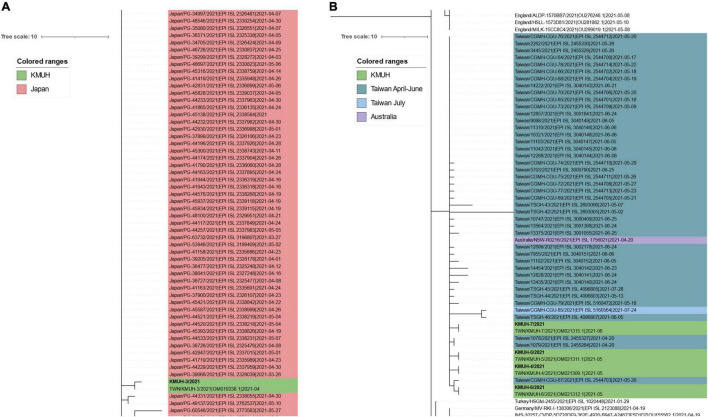
Phylogenetic analysis of KMUH-3 to KMUH-7 SARS-CoV-2 using UShER. UShER enables real-time sequence placement for the SARS-CoV-2 pandemic using an existing phylogenetic tree generated by the sarscov2phylo pipeline, which contains 6,624,590 genomes from GISAID, GenBank, COG-UK, and CNCB (2 January 2022). The phylogenetic subtree data are visualized using Interactive Tree Of Life (iTOL) version 6.4.3 (https://itol.embl.de/) (73). The 200 nearest neighboring GISAID EpiCoV and/or other publicly available SARS-CoV-2 sequences, including the sequences uploaded for analysis, already in the existing phylogenetic tree were output for visualization. Only partial results are shown in each subtree panel. **(A)** The 200 nearest neighboring sequences to the KMUH-3 are all retrieved from Japan. **(B)** The 200 nearest neighboring sequences to KMUH-4 to KMUH-7 were retrieved from Taiwan, Australia, and European countries.

To reveal the possible origin of the five SARS-CoV-2 sequences identified in this study, we performed real-time phylogenetic analysis using Ultrafast Sample placement with Existing tRee (UShER) version 6.4.3^9^ ^10^ (49) to find the most similar complete and high-coverage SARS-CoV-2 sequences from publicly available SARS-CoV-2 databases (e.g., GISAID, GenBank COG-UK, and CNCB) (the analysis was performed on 2 January 2022). The results suggested that KMUH-3 (KMUH-3/2021| EPI_ISL_5395633| 2021-04-11) is similar to the SARS-CoV-2 sequences collected between March and May in Japan (Figure 3A). The results for KMUH-4 to KMUH-7 (KMUH-4/2021| EPI_ISL_7016374| 2021-05-19, KMUH-5/2021| EPI_ISL_7016459| 2021-05-22, KMUH-6/2021| EPI_ISL_7016494| 2021-05-24, and KMUH-7/2021| EPI_ISL_7016498| 2021-06-29) clustered with other SARS-CoV-2 sequences collected between April and July in Taiwan, and they are similar to other SARS-CoV-2 sequences collected from many countries in Europe (Figure 3B). We also analyzed the possible origin of the five SARS-CoV-2 sequences using AudacityInstant,^11^ which searches the entire GISAID EpiCoV site to find related sequences, and the results were similar to those obtained by using UShER (data not shown).

### Clade Replacements of SARS-CoV-2 Identified in Taiwan Between 2020 and 2021

**FIGURE 4:**
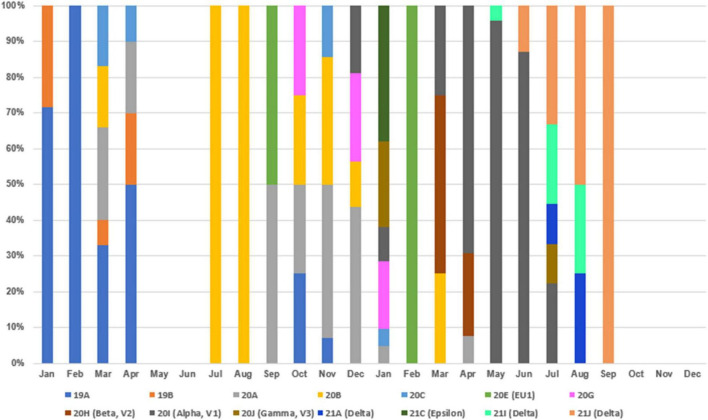
Clades replaced over time between January 2020 and December 2021 in Taiwan.

To understand the clade replacements of SARS-CoV-2 and the phylogenetic relationship among SARS-CoV-2 isolates identified in Taiwan between January 2020 and December 2021, we searched GISAID EpiCoV with Taiwan as a query in the field “Location” and downloaded SARS-CoV-2 genomes for further analysis on 2 January 2022. The query resulted in 267 sequences, including the five 20I (alpha, V1)/B.1.1.7/GR sequences identified in this study and two 19A/B/L isolates (KMUH-1 and KMUH-2) identified in our previous study (47). The replacement of SARS-CoV-2 clades over time between January 2020 and December 2021 in Taiwan is listed in Supplementary Table 3 and visualized in Figure 4. Clades 19A and 19B first emerged in January and February 2020 and then came the clades 20A/20B/20C in March and April 2020. With no COVID-19 cases in May and June, clade 20B reemerged in July and August, and clade 20A reemerged between September and December. Clades 20E and 20G first appeared in September and October but were not long-lived, suggesting that these COVID-19 cases were not responsible for large-scale community infections, with effective monitoring and isolation. With various VOCs dominating in 2021, clade replacements started from imported cases of 20I (alpha, V1)/B.1.1.7 isolates in December 2020, followed by clades 20J (gamma, V3), 21C (epsilon), B.1.351 (beta) and B.1.429 (epsilon) in January 2021, clades 21A (delta), 21I (delta), and 21J (delta) in July 2021, and clades AY.4.2 (delta plus) and B.1.1.529 (omicron) in December 2021 (special events from Daily COVID-19 Press Release by the CECC; see text footnote 2). The clade replacements in Taiwan in 2021 were similar to the time course of variant distribution for all submitted sequences in GISAID EpiCoV in 2021.^12^

### Phylogenetic Relationship for SARS-CoV-2 Identified in Taiwan Between 2020 and 2021

**FIGURE 5:**
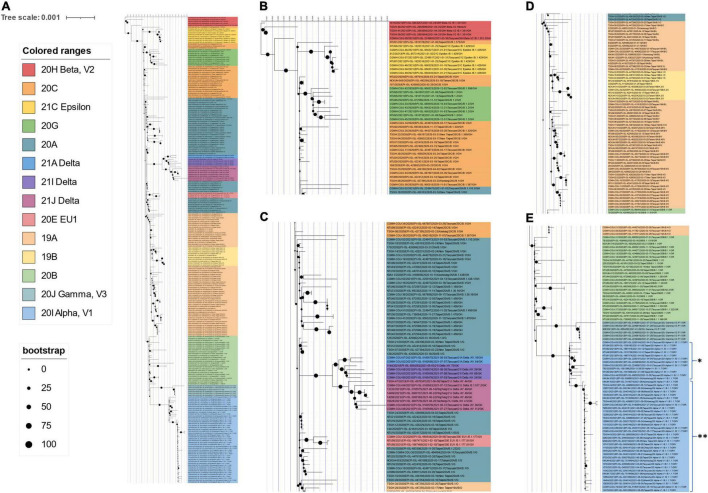
Phylogenetic tree of 247 SARS-CoV-2 genomes collected in Taiwan between January 2020 and December 2021. The phylogenetic analysis was inferred by using the maximum likelihood and fits of 286 different nucleotide substitution models, and the results suggested GTR + F + I as the best-fitting model with the lowest Bayesian information criterion (BIC) scores of 107,672.091 among the 286 models tested. The tree topology was automatically computed to estimate maximum likelihood values. The optimal log-likelihood for this computation was –50,857.073. There was a total of 29,867 positions in the final dataset. The original tree is displayed using iTOL v 6.4.3 (https://itol.embl.de/) (73) with an indicator of bootstrap values and a scale bar. Viruses are shown as the virus name| Accession ID| Collection date| City/Nextstrain_clade/pangolin_lineage/GISAID_clade). **(A)** An original phylogenetic tree of 247 SARS-CoV-2 sequences obtained from GISAID which samples were collected between January 2020 and December 2021 in Taiwan. **(B)** A partial tree exhibited phylogenetic correlation between Nextstrain clades 20H beta-V2, 20C, 21C epsilon, and 20G. **(C)** A partial tree exhibited phylogenetic correlation between Nextstrain clades 20A, 21A delta, 21I delta, 21J delta, and 20E EU1. **(D)** A partial tree exhibited phylogenetic correlation between Nextstrain clades 19A and 19B. **(E)** A partial tree exhibited phylogenetic correlation between Nextstrain clades 20B, 20J, and 20I alpha-V1. KMUH-3 was clustered with alpha/B.1.1.7 variant strain sequences without spike M1237I mutation collected between 26 December 2020 and 21 April 2021. KMUH-4 to KMUH-7 were clustered with alpha/B.1.1.7 variant strain sequences with spike M1237I mutation collected between 20 April 2021 and 28 July 2021. *Alpha/B.1.1.7 without spike M1237I mutation; ^**^alpha/B.1.1.7 with spike M1237I mutation.

For phylogenetic tree reconstruction, the 267 downloaded SARS-CoV-2 sequences were checked manually for sequences containing long runs of N (≥3), which were not included in the next step of the analysis, and a panel of 247 sequences was reserved for phylogenetic tree reconstruction. These 247 sequences were aligned using MAFFT v 7.490 (40); the sequences were trimmed at the 5′ and 3′ ends to produce the same size of genomic sequences (29,867 nucleotides). The most appropriate evolutionary model for reconstruction of the phylogenetic tree of these 247 SARS-CoV-2 genomes was analyzed by using ModelFinder (41). Evolutionary analyses were conducted in IQ-TREE 2.1.3 COVID-edition (42). An original tree of 247 SARS-CoV-2 sequences is depicted in Figure 5A; for the convenience of visualization, the tree is subdivided into panels (Figures 5B–E). The 247 sequences were classified into 14 phylogenetic clades and/or linages. The phylogenetic results suggest that the sequence of KMUH-3/2021| EPI_ISL_5395633 (GenBank OM019338) is most similar to CGMH-CGU-63/2021| EPI_ISL_2250184| 2021-04-21, CGMH-CGU-44/2020| EPI_ISL_956325| 2020-12-26, NTU52/2021| EPI_ISL_1041958| 2021-01-06, KMUH-4/2021| EPI_ISL_7016374 (GenBank OM021309), TSGH-44/2021| EPI_ISL_4096803| 2021-05-13, and 12857/2021| EPI_ISL_3001841| 2021-06-24. Additionally, KMUH-5/2021| EPI_ISL_7016459 (GenBank OM021311) is most similar to 13435/2021| EPI_ISL_3040140| 2021-06-24, 10321/2021| EPI_ISL_3040148| 2021-06-06, 12857/2021| EPI_ISL_3001841| 2021-06-24, and TSGH-44/2021| EPI_ISL_4096803| 2021-05-13. KMUH-6/2021| EPI_ISL_7016494 (GenBank OM021312) and KMUH-7/2021| EPI_ISL_7016498 (GenBank OM021315) are similar to some other sequences. The collection date and city suggest that the clade alpha/B.1.1.7 spread from cities in northern Taiwan (e.g., Taoyuan City and Taipei City) to central Taiwan (Changhua County and Miaoli County) and later to southern Taiwan (Kaohsiung City and Pingtung County) (Figure 5). Considering the collection date of the sequences deposited in the GISAID database, the clade replacements are in agreement with the news released by the CECC (Figure 5). These results illustrate the possible correlation between these sequences, not only for the five 20I (alpha, V1)/B.1.1.7/GR sequences identified in this study but also for the other SARS-CoV-2 sequences in the tree, as well as for the footprint of SARS-CoV-2 infection in Taiwan. The presence of a clade in a specific city at a specific time interval indicates that geographically related community infections may occur in COVID-19 patients infected with these viruses.

## Discussion

In this study, we isolated 4 SARS-CoV-2 strains from 13 nasopharyngeal swabs and identified 5 SARS-CoV-2 sequences during the largest COVID-19 outbreak in Taiwan between April and June 2021. According to the NGS results and *in silico* bioinformatics analysis, one of these viruses (KMUH-3) is of the 20I (alpha, V1)/B.1.1.7/GR lineage; the others (KMUH-4 to KMUH-7) belong to the 20I (alpha, V1)/B.1.1.7/GRY lineage, which was the VOC dominating worldwide at that time. These viruses share similar amino acid deletions (e.g., NSP6 3675–3677del, spike 69–70del, and spike 144del) and non-synonymous amino acid variations (e.g., spike N501Y, D614G, and P681H) predicted by using Dindel (v1.01) (36), Lofreq (v2.1.5) (37), GISAID and Nextclade v1.10.0. The SNV frequency and InDel frequency data suggest heterogeneity of KMUH-3, KMUH-6, and KMUH-7 in COVID-19 patients (Supplementary Table 2). In general, SARS-CoV-2 mutates rapidly even in a single individual. Notably, SARS-CoV-2 was only successfully isolated from nasopharyngeal swab-UTM with a Ct value ≤24 (e.g., samples 32, 36, 38, and 41), whereas other clinical samples with Ct values of 25.69 to 36.5 failed to produce virus. Some studies have shown that it is difficult to isolate SARS-CoV-2 from clinical samples with Ct > 30–35 because RT-PCR detects trace amounts of the genomic sequence by amplification of a target sequence but cannot discriminate genomic fragments from live infectious virus (50–55). In our recent study, SARS-CoV-2 (19A/B/L) was detected from 1/3 swabs with a Ct > 35 for the three genes; VERO E6 cells were used for virus culture with blind passage on day 3 when CPE was not observed (47), as blind passage usually results in increased virus infectivity and optimization of the isolation of slow-growing viruses (56, 57). It is possible that the failure in virus isolation was due to RNA degradation in these samples, as confirmed in the NGS process. Another reason why we did not successfully isolate SARS-CoV-2 from sample number 39 (Ct value: 26.42 for E gene and 25.69 for RdRp gene) and 40 (Ct value: 28.81 for E gene and 28.32 for RdRp gene) may be that the virus was inactivated during sample collection or processing, as the presence of a genome does not necessarily indicate a live virus in the sample (58). In addition, there are reports that no SARS-CoV-2 can be isolated by cell culture using clinical samples obtained from COVID-19 patients post-symptom onset (PSO) greater than eight days (51). This might be the reason why we isolated SARS-CoV-2 from samples 32, 36, 38, and 41 (PSO: 2–5 days) but not from other samples (PSO: 8–20 days).

To delineate the possible origin of the five SARS-CoV-2 sequences identified in this study, real-time sequence placement for the SARS-CoV-2 pandemic was performed using an existing phylogenetic tree generated by the sarscov2phylo pipeline, which contains 6,624,590 genomes from GISAID, GenBank, COG-UK, and CNCB. The phylogenetic subtree results suggest that KMUH-3 is closest to ∼200 SARS-CoV-2 sequences collected in Japan during their alpha/B.1.1.7 outbreak (59, 60) and that KMUH-4 to KMUH-7 are closest to several SARS-CoV-2 sequences collected in Taiwan during the largest alpha/B.1.1.7 outbreak (61). Patient number 32 (KMUH-3) had a traveling history to Japan before diagnosed of COVID-19 and patients number 36, 37, 38, and 41 (KMUH-4 to 7) had no traveling history abroad. The contact history and travel history of these COVID-19 patients validated the usage of UShER (49) to assess the feasibility of a certain virus from which viruses may originate or the same ancestors.

This alpha strain (B.1.1.7) outbreak ended in July. The epidemic was seamlessly integrated by the delta strain (21A, 21I, and 21J), continuing the outbreak into September. The situation in Taiwan usually started with cases imported from abroad (20, 26, 62), and approximately 93.2% of all confirmed cases in 2020 were imported. The largest COVID-19 outbreak ever in Taiwan occurred between May and June 2021 and resulted in 13,795 cases and 820 deaths, with a high CFR, at 5.95%, accounting for 80.90% of all cases and 96.47% of all deaths during the first 2 years of COVID-19 (Table 3). The distribution of age and sex of all recorded COVID-19 deaths between May and June 2021 is shown in Table 4. In general, the death rate was significantly higher in all male age-groups except for age group of 40–49 years old. The death rate was 63.17% in the group aged over 70 years old, which was significantly higher than other age groups (*P* = 0.008, Chi-square test). There were one or more comorbidities in 90.14% of all death cases, especially those in the group aged over 70 years old even up to 64.32%. The CFR in Taiwan was 2.8-fold that of the world (5.95 vs. 2.15%) between May and June 2021 (see text footnote 7): the rates for other countries, such as the United States, Germany, Israel, Vietnam, Japan, South Korea, and Singapore, were 2.19, 2.37, 2.00, 0.33, 2.20, 0.54, and 0.42%, respectively. Moreover, a recent meta-analysis including studies from 1 June 2020 to 15 October 2021, concluded that “Alpha, Beta, Gamma, and Delta variants are all more serious than the wild-type virus in terms of hospitalization, ICU admission, and mortality, and the Beta and Delta variants have a higher risk than the Alpha and Gamma variants.” The random effects of the beta and delta variants on the wild-type virus with respect to mortality rate are 1.50 (95% CI: 1.26–1.74) and 2.33 (95% CI: 1.45–3.21), respectively. The mortality rate of the alpha variant ranges 0.3–32.1% among studies (63). The unusually high CFR in Taiwan, a developed country, might be attributable to the following factors. First, 88–90% of death cases had one or more comorbidities (29) (Table 4). Second, low herd immunity resulted from the low prevalence of COVID-19 and low vaccination rate before the outbreak. The outbreak that started in mid-May boosted Taiwanese people’s willingness to get vaccinated which the vaccination program started on 22 March, the time when Taiwanese citizens were uncertain about the effects and side effects of vaccination. However, the first dose vaccination rate was only 6.9% (AstraZeneca or Moderna) among all citizens on 21 June 2021 (data were released on 22 June 2021^13^). In addition, according to the data released by Taiwan CECC, the seroprevalence of anti-N and anti-S antibody, which were induced by natural infection, was 0.02% (1/5,000) in serum samples from blood donations (donors were 17–65 years old) to the blood centers of the Taiwan Blood Services Foundation collected between 25 April and 3 July 2021. Moreover, seroprevalence of anti-S antibody, which induced by vaccination, was 5.2% (258/5,000) (data were released on 29 January 2022, see text footnote 13).

**TABLE 3:** COVID-19 cases between January 2020 and December 2021.^a^

Year	Month	Confirmed	Autochthonous	Imported	Death	Clade/Linage dominating^b^
2020	January	19	8	11	1	19A
	February	26	17	9	2	19A
	March	330	27	303	4	19A, 20A, 20B, 20C
	April	61	3	58	0	19A
	May	9	0	9	0	–
	June	6	0	6	0	–
	July	29	0	29	0	–
	August	17	0	17	0	–
	September	25	0	25	0	–
	October	53	0	53	0	–
	November	120	0	120	0	20A, 20B
	December	128	1	127	2	20A, 20G, alpha (G)(3)^c^
2020	January–December	823	56	767	9	Wild type
2021	January	114	19	95	2	Epsilon, gamma, 20G, alpha (G)(2)
	February	30	2	28	0	–
	March	87	0	87	0	Alpha (G)(1), beta (2)
	April	108	26	82	4	Alpha (G)(7), alpha (G + I)(2)^c^, beta (3)
	May	8,924	8,788	136	592	Alpha (G + I) (23), delta (1)
	June	4,871	4,831	40	228	Alpha (G + I) (20), delta (3)
	July	779	669	110	9	Alpha (G + I) (2), delta (6), gamma (1)
	August	316	149	167	5	Delta (4)
	September	208	41	167	1	Delta (1)
	October	166	9	157	0	–
	November	198	1	197	0	–
	December	426	9	417	0	–
2021	January–December	16,227	14,544	1,683	841	Alpha (G + I)

*^a^COVID-19 data were retrieved from the web-based notifiable disease surveillance system maintained by the TCDC. Source of data: https://nidss.cdc.gov.tw/nndss/disease?id=19CoV.*

*^b^According to the data deposited in GISAID EpiCoV. Refer to Supplementary Table 3 for full information.*

*^c^Alpha (G) refers to alpha/B.1.1.7 (D614G). Alpha (G + I) refers to alpha/B.1.1.7 (D614G + M1237I).*

**TABLE 4:** Distribution of the age and sex of confirmed COVID-19 patients who died between May and June 2021.^a^

Age	Gender	Death case number	Comorbidities	Total case number	Death percentage	*P*-value^b^
30–39	Male	7	5	1,026	0.68	0.038
	Female	1	0	988	0.10	
40–49	Male	6	6	916	0.66	0.221
	Female	14	9	1,190	1.18	
50–59	Male	50	45	1,161	4.30	<0.001
	Female	14	14	1,402	1.00	
60–69	Male	143	125	1,506	9.50	<0.001
	Female	67	60	1,284	5.22	
>70	Male	316	285	1,067	29.62	<0.001
	Female	202	191	880	22.95	
Total	Male	522	466	5,676	9.20	<0.001
	Female	298	274	5,744	5.19	

*^a^Source of data: http://at.cdc.tw.*

*^b^Chi-square test.*

**FIGURE 6:**
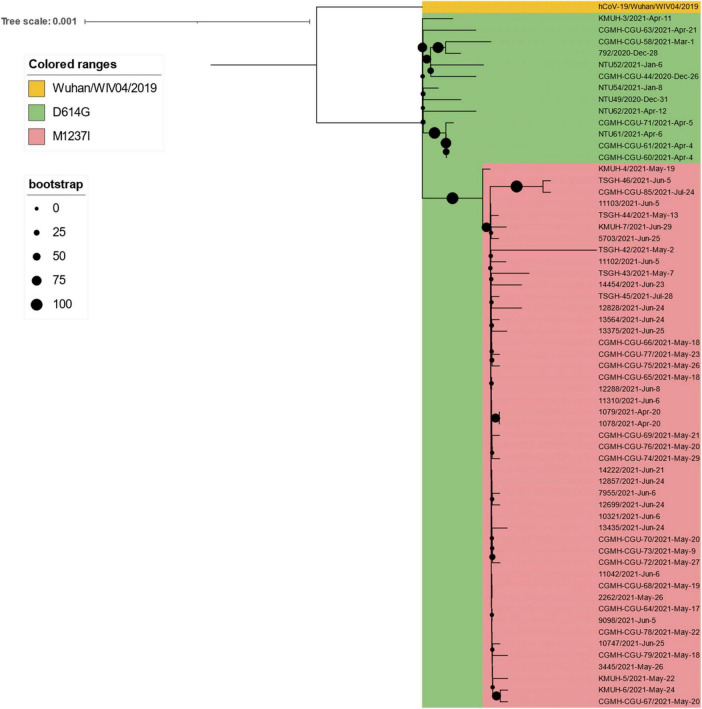
Phylogenetic tree of all 60 alpha/B.1.1.7 sequences deposited in GISAID EpiCoV from samples collected in Taiwan between January 2020 and December 2021. The phylogenetic analysis was inferred by using the maximum likelihood and fits of 286 different nucleotide substitution models, and the results suggested TN + F as the best-fitting model with the lowest Bayesian information criterion (BIC) scores of 85,071.899 among the 286 models tested. The tree topology was automatically computed to estimate the maximum likelihood values. The optimal log-likelihood for this computation was –42,000.232. There was a total of 29,796 positions in the final dataset. The original tree is displayed using iTOL v 6.4.3 (https://itol.embl.de/) (73) with an indicator of bootstrap values and a scale bar. Viruses are shown as the virus name| collection date.

Leung et al. suggested that the transmissibility of SARS-CoV-2 without 69–70del containing spike 501Y is ∼10% greater than that of the virus containing 501N and that the transmissibility of SARS-CoV-2 with 69–70del containing 501Y is 70–80% greater than that of the virus containing 501N (64). Their results also indicate that the G614 mutant is 28–34% more transmissible than the D614 wild-type (65). The SARS-CoV-2 alpha/B.1.1.7 variant featuring the above mutations, which was first detected in the United Kingdom, has increased transmissibility *via* enhanced spike RBD binding to the host ACE2 receptor and host immunity escape by abolishing its binding to the neutralizing antibody (66). According to GISAID data, alpha/B.1.1.7 entered Taiwan during the period from 26 December 2020 to January 2021. This information is consistent with the Taiwan government’s data (see text footnote 13). However, the above information does not explain why this wave of alpha/B.1.1.7 did not cause a COVID-19 outbreak similar to the one that occurred between May and June 2021. Because the earliest study that defined alpha/B.1.1.7 did not include the D614G mutation (67), we hypothesize that alpha/B.1.1.7 from 26 December 2020 to January 2021 may lack the D614G mutation; thus, its transmissibility was lower than that of the alpha/B.1.1.7 strain between May and June 2021. We aligned all 60 alpha/B.1.1.7 sequences deposited in GISAID EpiCoV, the samples for which were collected in Taiwan between January 2020 and December 2021, with the reference SARS-CoV-2 sequence (MN908947) and found that all 60 alpha/B.1.1.7 sequences carry the D614G mutation. Nevertheless, we found that 47 of the 48 alpha/B.1.1.7 sequences collected after 20 April possess the specific spike M1237I mutation, including KMUH-4 to KMUH-7. For the distribution of alpha/B.1.1.7 with or without spike M1237I mutation in different months, please refer to the right column “Clade/Linage dominating” of Table 3. These B.1.1.7 sequences with the spike M1237I mutation cluster in a subgroup in the phylogenetic tree, whereas those without the mutation cluster in another subgroup (Figures 5E, 6). In the subtree resulting from real-time sequence placement analysis using UShER, KMUH-4 to KMUH-7, which were collected between May and June 2021, cluster with other sequences collected in Taiwan between 20 April and 28 July 2021(Figure 3B); these sequences feature the M1237I mutation but do not cluster with the sequences without the M1237I mutation collected earlier in Taiwan. Amino acid 1,237 of spike protein is located at the junction between transmembrane domain (1,213–1,237 residues) and cytoplasm domain (1,237–1,273 residues) (68). The role of M1237I mutation alone in spike protein in SARS-CoV-2 was not reported before and largely unknown. According to the results of Li et al., the D614G + M1237I mutation decreases sensitivity to convalescent sera (69). This B.1.1.7 (D614G + M1237I) strain may lead to problems such as diagnosis and/or treatment failure (70, 71), which may be the reason why the B.1.1.7 (D614G + M1237I) epidemic between May and June 2021 was out of control despite similar prevention measures starting in 2020. In addition to the two possible causes of the high CFR in Taiwan, an unanswered question is whether the spike D614G and M1237I mutations and/or genetic diversity in Taiwanese individuals play a role in the high CFR during the alpha/B.1.1.7 outbreak. More studies need to be conducted to answer this question.

**FIGURE 7:**
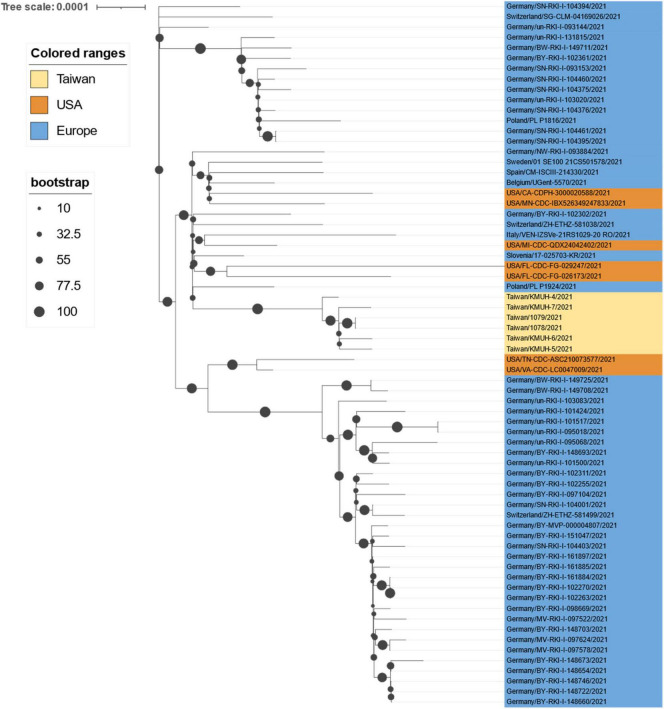
Phylogenetic analysis results of cases 1078/1079, KMUH-4 to KMUH-7, and 62 alpha/B.1.1.7 sequences with D614G and M1237I mutations deposited in GISAID from samples collected between 14 April 2021 and 16 April 2021. The phylogenetic analysis was inferred by using the maximum likelihood and fits of 286 different nucleotide substitution models, and the results suggested TN + F + I as the best-fitting model with the lowest BIC scores of 86,786.332 among the 286 models tested. The tree topology was automatically computed to estimate the maximum likelihood values. The optimal log-likelihood for this computation was –42,677.481. There was a total of 29,662 positions in the final dataset. The original tree is displayed using iTOL v 6.4.3 (https://itol.embl.de/) (73) with an indicator of bootstrap values and a scale bar. Viruses are shown as the virus name.

Although contact tracing was performed to clarify the correlation between COVID-19 cases during the early stage of this alpha/B.1.1.7 outbreak, there was always missing information that prevented several cluster infection events from being linked together. Furthermore, the source of the virus in the outbreak has been controversial. According to the daily COVID-19 Press Release by the CECC, there were only two confirmed COVID-19 cases (case numbers 1,078 and 1,079) in Taiwan on 20 April 2021. Notably, the earliest sequences containing spike M1237I deposited in GISAID and collected in Taiwan were 1078/2021| EPI_ISL_2455327| 2021-04-20 and 1079/2021| EPI_ISL_2455264| 2021-04-20, with age and sex matching, which were deposited by the TCDC. These two COVID-19 cases were the cargo crew members of China Airlines, who flew to the United States with their colleagues on 14 April for duty and performed the stay-at-home notice at a local hotel until returning to Taiwan on 16 April. The two crew members developed symptoms on 17 and 18 April. We retrieved sequences with M1237I mutation collected between 14 April 2021 and 16 April 2021 from GISAID. Phylogenetic analysis was performed using a set of sequences containing cases 1,078, 1,079, KMUH-4 to KMUH-7 and other 62 alpha/B.1.1.7 sequences with M1237I mutation from GISAID. The 68 sequences were all featured with D614G and M1237I mutations. The results suggested that cases 1,078, 1,079, KMUH-4 to KMUH-7 were phylogenetically highly close to the sequences collected from United States, Poland and Slovenia (Figure 7). Based on the traveling history of cases 1,078 and 1,079, it is likely that these two crew members were infected in the United States or got infected from United States or European travelers in the airplane. Taking the information revealed in the COVID-19 Press Release by the CECC, GISAID and the molecular evidences described above, we suggest that the largest COVID-19 outbreak ever in Taiwan history started from the alpha/B.1.1.7 (D614G + M1237I) cluster infection event occurring at China Airlines, whereby cargo crew members were infected during their task.

There are some limitations in this study. First, although we successfully isolated SARS-CoV-2 by using VERO E6 cell culture, the usage of genetically modified cells (e.g., ACE2- and TMPRSS2-overexpressing cells) might increase binding of the virus to target cells (13), increasing the opportunity for isolation. Second, we isolated SARS-CoV-2 using VERO E6 cells incubated at 37°C, which is the temperature most researchers use (18, 52–54). However, incubation at lower temperatures (e.g., 32–34°C) might enhance the chance of isolating SARS-CoV-2 because the virus spreads primarily through active virus shedding from the nasopharynx, a body site at which the temperature is lower than 37°C (14, 56, 57, 72). Third, the COVID-19 epidemic news released by the Taiwanese government in 2020 does not contain information such as linage and/or clade. In addition, the sequence downloaded from the GISAID database may not fully represent the full picture of the epidemic virus strain at that time. Nevertheless, the results of clade replacement analysis for Taiwan’s COVID-19 epidemic between January 2020 and December 2021 in this study are the most complete thus far. Finally, the results of phylogenetic analysis of the 247 sequences isolated in Taiwan explains the distance between them but does not indicate the evolutionary relationship because this virus accumulates genetic variations very quickly and no travel history and/or gathering history are available in the sequence repository, even though information regarding the sample collection date and city is included as part of the sequence identity.

## Conclusion

In this study, we isolated four 20I (alpha, V1)/B.1.1.7/GRY SARS-CoV-2 strains by using VERO E6 cell culture and identified five SARS-CoV-2 sequences from COVID-19 patients in the largest COVID-19 outbreak in Taiwan between April and June 2021. Sequence placement analysis of the existing SARS-CoV-2 phylogenetic tree by using UShER revealed that KMUH-3 originated from Japan and KMUH-4 to KMUH-7 possibly through local transmission. We conclude that the largest COVID-19 outbreak in Taiwan between May and June 2021 was initially caused by the alpha/B.1.1.7 variant containing spike D614G + M1237I mutations, which was introduced to Taiwan by cargo crew members of China Airlines. The largest COVID-19 outbreak in Taiwan resulted in 13,795 cases and 820 deaths, with a 5.95% CFR, accounting for 80.90% of all cases and 96.47% of all deaths during the first 2 years of COVID-19. The high CFR caused by SARS-CoV-2 alpha variants in Taiwan can be attributable to comorbidities and low herd immunity. We also suggest that SARS-CoV-2 isolation and sequencing of isolates in a timely manner are of great importance in real-time epidemiological investigations and epidemic prevention. The impact of D614G + M1237I mutations in the spike gene on the SARS-CoV-2 virus spreading as well as on high CFR remains to be elucidated.

## Data Availability Statement

The datasets presented in this study can be found in online repositories. The names of the repository/repositories and accession number(s) can be found below: https://www.ncbi.nlm.nih.gov/genbank/, OM019338, OM021309, OM021311, OM021312, and OM021315.

## Ethics Statement

The studies involving human participants were reviewed and approved by the Institutional Review Board of Kaohsiung Medical University Hospital. The patients/participants provided their written informed consent to participate in this study.

## Author Contributions

J-JT, C-HC, and I-WC: resources. J-JT, KC, P-CL, W-LC, J-MC, S-JH, and I-WC: investigation. J-JT, C-HC, P-CL, C-YT, Y-YT, and M-CH: methodology. L-TL, KC, P-CL, C-YT, and Y-YT: data curation. J-JT, P-CL, and C-HC: conceptualization. J-JT, C-HC, W-LC, J-MC, S-JH, and I-WC: supervision. L-TL, J-JT, C-HC, W-LC, J-MC, S-JH, and I-WC: validation. L-TL, J-JT, and P-CL: writing—original draft. L-TL and J-JT: writing—review and editing. All authors contributed to the article and approved the submitted version.

## Conflict of Interest

The authors declare that the research was conducted in the absence of any commercial or financial relationships that could be construed as a potential conflict of interest.

## Publisher’s Note

All claims expressed in this article are solely those of the authors and do not necessarily represent those of their affiliated organizations, or those of the publisher, the editors and the reviewers. Any product that may be evaluated in this article, or claim that may be made by its manufacturer, is not guaranteed or endorsed by the publisher.

## Footnotes

1
https://www.gisaid.org/

2
http://at.cdc.tw

3
https://github.com/artic-network/artic-ncov2019/tree/master/primer_schemes/nCoV-2019/V3

4
https://artic.network/

5
https://mafft.cbrc.jp/alignment/software/

6
https://nidss.cdc.gov.tw/nndss/disease?id=19CoV

7 https://covid.ourworldindata.org/data/owid-covid-data.xlsx, data were accessed on 1 January 2022.

8
https://imdataman.github.io/mapbox-density-map/

9
https://clades.nextstrain.org

10
https://genome.ucsc.edu/cgi-bin/hgPhyloPlace

11
https://www.epicov.org/epi3/frontend#408fa4

12
https://www.epicov.org/epi3/frontend#lightbox1829923246

13
https://www.cdc.gov.tw/

## Supplementary Material

Supplementary Figure 1 Cumulative confirmed COVID-19 cases from 22 January 2020 to 31 December 2021. Hannah Ritchie, Edouard Mathieu, Lucas Rodés-Guirao, Cameron Appel, Charlie Giattino, Esteban Ortiz-Ospina, Joe Hasell, Bobbie Macdonald, Diana Beltekian, and Max Roser (2020) – “Coronavirus Pandemic (COVID-19).” Published online at OurWorldInData.org. Retrieved from: “https://ourworldindata.org/explorers/coronavirus-data-explorer?tab=map&zoom ToSelection=true&time=2021-12-31&facet=none&hideControls=true&Metric= Confirmed+cases&Interval=Cumulative&Relative+to+Population=false&Color+ by+test+positivity=false&country=∼OWID_WRL” (online resource).

Supplementary Figure 2 A presentation of NGS coverage, depth and SNVs of SARS-CoV-2. **(A)** NGS coverage and depth of KMUH-3 (RNA-seq) and KMUH-5 (targeted sequencing) according to the genomic positions of the reference strain Wuhan-Hu-1/2019 (MN908947). **(B)** SNVs at nt positions 14,676 (NSP12 P4804P), 23,063 (spike N501Y), and 27,513 (NS7a Y40Y) were confirmed by Sanger sequencing (KMUH-4).

Supplementary Figure 3 Accumulating imported COVID-19 cases from different countries between January 2020 and December 2021 in Taiwan. Source of data: https://nidss.cdc.gov.tw/nndss/disease?id=19CoV.
