# Corrigendum: Identification and analysis of SARS-CoV-2 alpha variants in the largest Taiwan COVID-19 outbreak in 2021

**DOI:** 10.3389/fmed.2023.1211944

**Published:** 2023-05-17

**Authors:** Li-Teh Liu, Jih-Jin Tsai, Ko Chang, Chun-Hong Chen, Ping-Chang Lin, Ching-Yi Tsai, Yan-Yi Tsai, Miao-Chen Hsu, Wan-Long Chuang, Jer-Ming Chang, Shang-Jyh Hwang, Inn-Wen Chong

**Affiliations:** ^1^Department of Medical Laboratory Science and Biotechnology, College of Medical Technology, Chung Hwa University of Medical Technology, Tainan, Taiwan; ^2^Tropical Medicine Center, Kaohsiung Medical University Hospital, Kaohsiung, Taiwan; ^3^Division of Infectious Diseases, Department of Internal Medicine, Kaohsiung Medical University Hospital, Kaohsiung, Taiwan; ^4^School of Medicine, College of Medicine, Kaohsiung Medical University, Kaohsiung, Taiwan; ^5^Department of Internal Medicine, Kaohsiung Municipal Siaogang Hospital, Kaohsiung Medical University, Kaohsiung, Taiwan; ^6^National Mosquito-Borne Diseases Control Research Center, National Health Research Institutes, Zhunan, Taiwan; ^7^National Institute of Infectious Diseases and Vaccinology, National Health Research Institutes, Zhunan, Taiwan; ^8^Division of Hepatobiliary and Pancreatic, Kaohsiung Medical University Hospital, Kaohsiung, Taiwan; ^9^Division of Nephrology, Department of Internal Medicine, Kaohsiung Medical University Hospital, Kaohsiung, Taiwan; ^10^Department of Medical Research, Kaohsiung Medical University Hospital, Kaohsiung, Taiwan; ^11^Department of Internal Medicine and Graduate Institute of Medicine, Kaohsiung Medical University, Kaohsiung, Taiwan; ^12^Department of Pulmonary Medicine, Kaohsiung Medical University Hospital, Kaohsiung, Taiwan

**Keywords:** COVID-19, SARS-CoV-2, qRT-PCR, virus culture, next-generation sequencing, clade replacements, phylogenetic analysis, alpha/B.1.1.7

In the published article, there was an error. “D614G” was incorrectly written as “G614G” in the Abstract.

A correction has been made to the **Abstract**. This section previously stated:

“Severe acute respiratory syndrome coronavirus 2 (SARS-CoV-2) is believed to have originated in Wuhan City, Hubei Province, China, in December 2019. Infection with this highly dangerous human-infecting coronavirus *via* inhalation of respiratory droplets from SARS-CoV-2 carriers results in coronavirus disease 2019 (COVID-19), which features clinical symptoms such as fever, dry cough, shortness of breath, and life-threatening pneumonia. Several COVID-19 waves arose in Taiwan from January 2020 to March 2021, with the largest outbreak ever having a high case fatality rate (CFR) (5.95%) between May and June 2021. In this study, we identified five 20I (alpha, V1)/B.1.1.7/GR SARS-CoV-2 (KMUH-3 to 7) lineage viruses from COVID-19 patients in this largest COVID-19 outbreak. Sequence placement analysis using the existing SARS-CoV-2 phylogenetic tree revealed that KMUH-3 originated from Japan and that KMUH-4 to KMUH-7 possibly originated *via* local transmission. Spike mutations M1237I and D614G were identified in KMUH-4 to KMUH-7 as well as in 43 other alpha/B.1.1.7 sequences of 48 alpha/B.1.1.7 sequences deposited in GISAID derived from clinical samples collected in Taiwan between 20 April and July. However, M1237I mutation was not observed in the other 12 alpha/B.1.1.7 sequences collected between 26 December 2020, and 12 April 2021. We conclude that the largest COVID-19 outbreak in Taiwan between May and June 2021 was initially caused by the alpha/B.1.1.7 variant harboring spike D614G + M1237I mutations, which was introduced to Taiwan by China Airlines cargo crew members. To our knowledge, this is the first documented COVID-19 outbreak caused by alpha/B.1.1.7 variant harboring spike M1237I mutation thus far. The largest COVID-19 outbreak in Taiwan resulted in 13,795 cases and 820 deaths, with a high CFR, at 5.95%, accounting for 80.90% of all cases and 96.47% of all deaths during the first 2 years. The high CFR caused by SARS-CoV-2 alpha variants in Taiwan can be attributable to comorbidities and low herd immunity. We also suggest that timely SARS-CoV-2 isolation and/or sequencing are of importance in real-time epidemiological investigations and in epidemic prevention. The impact of G614G + M1237I mutations in the spike gene on the SARS-CoV-2 virus spreading as well as on high CFR remains to be elucidated.”

The corrected section appears below:

“Severe acute respiratory syndrome coronavirus 2 (SARS-CoV-2) is believed to have originated in Wuhan City, Hubei Province, China, in December 2019. Infection with this highly dangerous human-infecting coronavirus *via* inhalation of respiratory droplets from SARS-CoV-2 carriers results in coronavirus disease 2019 (COVID-19), which features clinical symptoms such as fever, dry cough, shortness of breath, and life-threatening pneumonia. Several COVID-19 waves arose in Taiwan from January 2020 to March 2021, with the largest outbreak ever having a high case fatality rate (CFR) (5.95%) between May and June 2021. In this study, we identified five 20I (alpha, V1)/B.1.1.7/GR SARS-CoV-2 (KMUH-3 to 7) lineage viruses from COVID-19 patients in this largest COVID-19 outbreak. Sequence placement analysis using the existing SARS-CoV-2 phylogenetic tree revealed that KMUH-3 originated from Japan and that KMUH-4 to KMUH-7 possibly originated *via* local transmission. Spike mutations M1237I and D614G were identified in KMUH-4 to KMUH-7 as well as in 43 other alpha/B.1.1.7 sequences of 48 alpha/B.1.1.7 sequences deposited in GISAID derived from clinical samples collected in Taiwan between 20 April and July. However, M1237I mutation was not observed in the other 12 alpha/B.1.1.7 sequences collected between 26 December 2020, and 12 April 2021. We conclude that the largest COVID-19 outbreak in Taiwan between May and June 2021 was initially caused by the alpha/B.1.1.7 variant harboring spike D614G + M1237I mutations, which was introduced to Taiwan by China Airlines cargo crew members. To our knowledge, this is the first documented COVID-19 outbreak caused by alpha/B.1.1.7 variant harboring spike M1237I mutation thus far. The largest COVID-19 outbreak in Taiwan resulted in 13,795 cases and 820 deaths, with a high CFR, at 5.95%, accounting for 80.90% of all cases and 96.47% of all deaths during the first 2 years. The high CFR caused by SARS-CoV-2 alpha variants in Taiwan can be attributable to comorbidities and low herd immunity. We also suggest that timely SARS-CoV-2 isolation and/or sequencing are of importance in real-time epidemiological investigations and in epidemic prevention. The impact of D614G + M1237I mutations in the spike gene on the SARS-CoV-2 virus spreading as well as on high CFR remains to be elucidated.”

In the published article, there was an error. “D614G” was incorrectly written as “G614G” in the Conclusion.

A correction has been made to the **Conclusion**. This section previously stated:

“In this study, we isolated four 20I (alpha, V1)/B.1.1.7/GRY SARS-CoV-2 strains by using VERO E6 cell culture and identified five SARS-CoV-2 sequences from COVID-19 patients in the largest COVID-19 outbreak in Taiwan between April and June 2021. Sequence placement analysis of the existing SARS-CoV-2 phylogenetic tree by using UShER revealed that KMUH-3 originated from Japan and KMUH-4 to KMUH-7 possibly through local transmission. We conclude that the largest COVID-19 outbreak in Taiwan between May and June 2021 was initially caused by the alpha/B.1.1.7 variant containing spike D614G + M1237I mutations, which was introduced to Taiwan by cargo crew members of China Airlines. The largest COVID-19 outbreak in Taiwan resulted in 13,795 cases and 820 deaths, with a 5.95% CFR, accounting for 80.90% of all cases and 96.47% of all deaths during the first 2 years of COVID-19. The high CFR caused by SARS-CoV-2 alpha variants in Taiwan can be attributable to comorbidities and low herd immunity. We also suggest that SARS-CoV-2 isolation and sequencing of isolates in a timely manner are of great importance in real-time epidemiological investigations and epidemic prevention. The impact of G614G + M1237I mutations in the spike gene on the SARS-CoV-2 virus spreading as well as on high CFR remains to be elucidated.”

The corrected section appears below:

“In this study, we isolated four 20I (alpha, V1)/B.1.1.7/GRY SARS-CoV-2 strains by using VERO E6 cell culture and identified five SARS-CoV-2 sequences from COVID-19 patients in the largest COVID-19 outbreak in Taiwan between April and June 2021. Sequence placement analysis of the existing SARS-CoV-2 phylogenetic tree by using UShER revealed that KMUH-3 originated from Japan and KMUH-4 to KMUH-7 possibly through local transmission. We conclude that the largest COVID-19 outbreak in Taiwan between May and June 2021 was initially caused by the alpha/B.1.1.7 variant containing spike D614G + M1237I mutations, which was introduced to Taiwan by cargo crew members of China Airlines. The largest COVID-19 outbreak in Taiwan resulted in 13,795 cases and 820 deaths, with a 5.95% CFR, accounting for 80.90% of all cases and 96.47% of all deaths during the first 2 years of COVID-19. The high CFR caused by SARS-CoV-2 alpha variants in Taiwan can be attributable to comorbidities and low herd immunity. We also suggest that SARS-CoV-2 isolation and sequencing of isolates in a timely manner are of great importance in real-time epidemiological investigations and epidemic prevention. The impact of D614G + M1237I mutations in the spike gene on the SARS-CoV-2 virus spreading as well as on high CFR remains to be elucidated.”

The authors apologize for these errors and state that this does not change the scientific conclusions of the article in any way. The original article has been updated.

