# Supplementary material for: Identification and Analysis of SARS-CoV-2 Alpha Variants in the Largest Taiwan COVID-19 Outbreak in 2021
Source: Front Med (Lausanne). 2022 Apr 25;9:869818. doi: 10.3389/fmed.2022.869818 (PMC9081839; doi:10.3389/fmed.2022.869818)
Supplement: Supplementary file 1 [file Table_1.doc]

**SUPPLEMENTARY TABLE 1 |** qRT–PCR primers, probes, mixture, machine and cycling conditions.

**A. Primers and probes used in SARS-CoV-2 qRT-PCR.**

| E_ Sarbeco_F1 Forward primer | 5’-ACAGGTACGTTAATAGTTAATAGCGT-3’ |
| --- | --- |
| E_Sarbeco_R2 Reverse primer | 5’-ATATTGCAGCAGTACGCACACA-3’ |
| E_Sarbeco_P1 Probe | 5’-FAM-ACACTAGCCATCCTTACTGCGCTTCG-BBQ-3’ |
| N_ Sarbeco_F1 Forward primer | 5’-CACATTGGCACCCGCAATC-3’ |
| N _Sarbeco_R1 Reverse primer | 5’-GAGGAACGAGAAGAGGCTTG-3’ |
| N_Sarbeco_P1 Probe | 5’-FAM-ACTTCCTCAAGGAACAACATTGCCA-BBQ-3’ |
| RdRp_SARSr-F2 Forward primer | 5’-GTGARATGGTCATGTGTGGCGG-3’ |
| RdRp_SARSr_R1 Reverse primer | 5’-CARATGTTAAASACACTATTAGCATA-3’ |
| RdRp_SARSr-P2 Probe | 5’-FAM-CAGGTGGAACCTCATCAGGAGATGC-BBQ-3’ |
| RdRp_SARSr-P1 Probe | 5’-FAM-CCAGGTGGWACRTCATCMGGTGATGC-BBQ-3’ |

**B.** qRT-PCR mixture composition.

LightCycler® Multiplex RNA Virus Master kit (Roche Diagnostics, Mannheim, Germany. Catalog number: 6754155001)

**For E and N genes**

| Components | Volume  µL |
| --- | --- |
| RT Enzyme Solution | 0.1 |
| RT-qPCR Reaction Mix | 4 |
| Water, PCR Grade | 8.4 |
| Forward Primer (10μM) | 1 |
| Reverse Primer (10μM) | 1 |
| Probe (5μM) | 0.5 |
| Template RNA | 5 |
| Total Volume | 20 |

For RdRP gene

| Components | Volume µL |
| --- | --- |
| RT Enzyme Solution | 0.1 |
| RT-qPCR Reaction Mix | 4 |
| Water, PCR Grade | 7.3 |
| Forward Primer (10μM) | 1.2 |
| Reverse Primer (10μM) | 1.6 |
| Probe (5μM) | 0.8 |
| Template RNA | 5 |
| Total Volume | 20 |

**C.** qRT-PCR cycling conditions.

PCR thermal cycler Mx3000P (Agilent, USA)

| Cycles | Temperature | Time | Step |
| --- | --- | --- | --- |
| 1x | 50 ^0^C | 10 min | Reverse Transcription |
| 1x | 95 ^0^C | 30 sec | Denaturation |
| 45x | 95 ^0^C | 5 sec | Denaturation |
|  | 53 ^0^C | 15 sec | Annealing |
|  | 60 ^0^C | 15 sec | Extension |

**D. Custom-designed primers and amplicon length for Targeted sequencing (multiplexed PCR) of low viral loads samples.**

| Position | Primer Name | Primer sequence (5'-3') | Amplicon (bp) |
| --- | --- | --- | --- |
| 142-296 | nCov-2019_PL3_1F_155bp | AATTACTGTCGTTGACAGGACACG | 155 |
|  | nCov-2019_PL3_1R_155bp | TCTCGTTGAAACCAGGGACAA |  |
| 442-600 | nCov-2019_PL3_2F_159bp | CGTTTTGCCTCAACTTGAACAG | 159 |
|  | nCov-2019_PL3_2R_159bp | CCCACATGAGGGACAAGGAC |  |
| 764-916 | nCov-2019_PL3_3F_153bp | AGTGGTGTTACCCGTGAACTCA | 153 |
|  | nCov-2019_PL3_3R_153bp | TTCGGACAAAGTGCATGAAGC |  |
| 1061-1214 | nCov-2019_PL3_4F_154bp | TTCAATGGGGAATGTCCAAATT | 154 |
|  | nCov-2019_PL3_4R_154bp | GGCACATTTGGTTGCATTCA |  |
| 1365-1527 | nCov-2019_PL3_5F_163bp | AAATTTATTGTCCAGCATGTCACAA | 163 |
|  | nCov-2019_PL3_5R_163bp | GCACACTTGTTATGGCAACCAA |  |
| 1685-1843 | nCov-2019_PL3_6F_159bp | GCCATTATTTTGGCATCTTTTTCT | 159 |
|  | nCov-2019_PL3_6R_159bp | CTGTTCACCAATATTCCAGGCA |  |
| 1988-2145 | nCov-2019_PL3_7F_158bp | CAGTATTCACTGAGACTCATTGATGC | 158 |
|  | nCov-2019_PL3_7R_158bp | ACGGGTTTGAGTTTTTCATAAACAG |  |
| 2283-2469 | nCov-2019_PL3_8F_187bp | AGAGTGTTCAGACATTCTTTAAGCTTGT | 187 |
|  | nCov-2019_PL3_8R_187bp | GCTTTTAGAGGCATGAGTAGGCC |  |
| 2626-2783 | nCov-2019_PL3_9F_158bp | GCTTATGTTGCTCGAAATCAAAGAC | 158 |
|  | nCov-2019_PL3_9R_158bp | TCACACTCTTGTAACCTTGCACTTCTA |  |
| 2941-3095 | nCov-2019_PL3_10F_155bp | ACTGGGCATTGATTTAGATGAGTG | 155 |
|  | nCov-2019_PL3_10R_155bp | ATGGCTCAAACTCTTCTTCTTCACA |  |
| 3263-3422 | nCov-2019_PL3_11F_160bp | ACTACTATTCAAACAATTGTTGAGGTTCA | 160 |
|  | nCov-2019_PL3_11R_160bp | TTACCTTTTTAGCTTCTTCCACAATGT |  |
| 3581-3734 | nCov-2019_PL3_12F_154bp | AGCGGACACAATCTTGCTAAACA | 154 |
|  | nCov-2019_PL3_12R_154bp | CAGCACCAAAAATACCAGCTGAT |  |
| 3890-4060 | nCov-2019_PL3_13F_171bp | GAGGAAGTTAAGCCATTTATAACTGAAAG | 171 |
|  | nCov-2019_PL3_13R_171bp | TGGATGAAGATTGCCATTAATGTC |  |
| 4145-4303 | nCov-2019_PL3_14F_159bp | GGTGTTTTAACTGCTGTGGTTATACC | 159 |
|  | nCov-2019_PL3_14R_159bp | AAGCACTGTCTTTGCCTCCTCTA |  |
| 4419-4572 | nCov-2019_PL3_15F_154bp | CACGCAAATTAATGCCTGTCTG | 154 |
|  | nCov-2019_PL3_15R_154bp | TTGATAAGTGACGCTACAGTTGTTTTAC |  |
| 4752-4907 | nCov-2019_PL3_16F_156bp | CACCTGAAGAACATTTTATTGAAACC | 156 |
|  | nCov-2019_PL3_16R_156bp | CATCTAGGTGGAATGTGGTAGGATT |  |
| 5054-5209 | nCov-2019_PL3_17F_156bp | ACTTATTTGGATGGAGCTGATGTTACT | 156 |
|  | nCov-2019_PL3_17R_156bp | TGACATGTACCTACCCAGAAAACTAGG |  |
| 5376-5529 | nCov-2019_PL3_18F_154bp | CTGGTGAAGCTGCTAACTTTTGTG | 154 |
|  | nCov-2019_PL3_18R_154bp | CAAGTTTTACACACCACGTTCAAGA |  |
| 5681-5838 | nCov-2019_PL3_19F_158bp | ATGATGTCAGCACCACCTGCT | 158 |
|  | nCov-2019_PL3_19R_158bp | TCTGAGGACTTTGTAAGTAAAGCACC |  |
| 5991-6150 | nCov-2019_PL3_20F_160bp | AGCAACCAATTGATCTTGTACCAA | 160 |
|  | nCov-2019_PL3_20R_160bp | TCAGGGAAAAATGTAACTTTAAGCTCT |  |
| 6288-6445 | nCov-2019_PL3_21F_158bp | GGTGTATACGTTGTCTTTGGAGCA | 158 |
|  | nCov-2019_PL3_21R_158bp | GTCTTTCTGTATGGTAGGATTTTCCAC |  |
| 6553-6753 | nCov-2019_PL3_22F_201bp | GGCTGCTTATGTAGACAATTCTAGTCTT | 201 |
|  | nCov-2019_PL3_22R_201bp | CGGTTTAAACACCGTGTAACTATGTT |  |
| 6838-7000 | nCov-2019_PL3_23F_163bp | AGCATCTATGCCGACTACTATAGCAA | 163 |
|  | nCov-2019_PL3_23R_163bp | GTAGATTAAAGAACCTAGGCAAACACTTAA |  |
| 7144-7300 | nCov-2019_PL3_24F_157bp | TGGTTTAGATTCTTTAGACACCTATCCTT | 157 |
|  | nCov-2019_PL3_24R_157bp | TGCAGCCAATCCAAGTACATAGA |  |
| 7405-7578 | nCov-2019_PL3_25F_174bp | GGTTAGAATGTACATCTTCTTTGCATCA | 174 |
|  | nCov-2019_PL3_25R_174bp | GCATAGACATAAAAGGACCTTCTAACAC |  |
| 7747-7921 | nCov-2019_PL3_26F_175bp | TACAGTGAAGAATGGTTCCATCCA | 175 |
|  | nCov-2019_PL3_26R_175bp | TGATTCTTCACATTTTGATTTACCATC |  |
| 8060-8226 | nCov-2019_PL3_27F_167bp | TCATCAACTTTTAACGTACCAATGGA | 167 |
|  | nCov-2019_PL3_27R_167bp | CATTCAACAACATCTTTAGTTTCTACATCT |  |
| 8372-8542 | nCov-2019_PL3_28F_171bp | GTAGCAAAAAGTCACAACATTGCTTT | 171 |
|  | nCov-2019_PL3_28R_171bp | TGCTATCTTTGTTGTTACAACATTAACAAC |  |
| 8713-8866 | nCov-2019_PL3_29F_154bp | CACTCGTGACATAGCATCTACAGATACT | 154 |
|  | nCov-2019_PL3_29R_154bp | GCACGACAAAACCCACTTCTCT |  |
| 9010-9162 | nCov-2019_PL3_30F_153bp | GGCTGCTGAATGTACAATTTTTAAAG | 153 |
|  | nCov-2019_PL3_30R_153bp | TTAGGAAATTGAATAATAGAGCCATCC |  |
| 9307-9465 | nCoV-2019_PL3_31F_159bp | ACCAGGAGTTTTCTGTGGTGTAG | 159 |
|  | nCoV-2019_PL3_31R_159bp | AACCTCATAAAATAGTAGGCAAGGC |  |
| 9558-9736 | nCoV-2019_PL3_32F_179bp | ACTCATTCTTACCTGGTGTTTATTCTG | 179 |
|  | nCoV-2019_PL3_32R_179bp | AAAGAACCAATAGAAATGCTTTGTGG |  |
| 9903-10058 | nCoV-2019_PL3_33F_156bp | AGTACAAGTATTTTAGTGGAGCAATGG | 156 |
|  | nCoV-2019_PL3_33R_156bp | CACTCTGCAAAACAGCTGAGG |  |
| 10187-10341 | nCoV-2019_PL3_34F_155bp | ACCTCTGAAGACATGCTTAACCC | 155 |
|  | nCoV-2019_PL3_34R_155bp | GGATTGGCTGTATCAACCTTAAGC |  |
| 10480-10638 | nCoV-2019_PL3_35F_159bp | TGGTTCATGTGGTAGTGTTGGT | 159 |
|  | nCoV-2019_PL3_35R_159bp | CCAGCTGCTTGTGCTGTTTG |  |
| 10787-10960 | nCoV-2019_PL3_36F_174bp | GACCATGTTGACATACTAGGACCTC | 174 |
|  | nCoV-2019_PL3_36R_174bp | ACCTGAGCATTGTCTAACAACATC |  |
| 11108-11281 | nCoV-2019_PL3_37F_174bp | GCTATGGGTATTATTGCTATGTCTGC | 174 |
|  | nCoV-2019_PL3_37R_174bp | AGTATCAACCATATCCAACCATGTC |  |
| 11393-11575 | nCoV-2019_PL3_38F_183bp | ACACTTATGAATGTCTTGACACTCG | 183 |
|  | nCoV-2019_PL3_38R_183bp | GAAGAAAATAGGGCAATACTCAACACA |  |
| 11664-11840 | nCoV-2019_PL3_39F_177bp | TCAACCGCTACTTTAGACTGACTC | 177 |
|  | nCoV-2019_PL3_39R_177bp | GTACAGTGGCTACTTTGATACAAGG |  |
| 11984-12138 | nCoV-2019_PL3_40F_155bp | GCCTTTGAAAAAATGGTTTCACTACT | 155 |
|  | nCoV-2019_PL3_40R_155bp | GCAAAAGCTGCATATGATGGAAGG |  |
| 12232-12370 | nCoV-2019_PL3_41F_139bp | TGAATTTGACCGTGATGCAGC | 139 |
|  | nCoV-2019_PL3_41R_139bp | AGTGAAAAGCATTGTCTGCATAGC |  |
| 12528-12685 | nCoV-2019_PL3_42F_158bp | CATTTACTTATGCATCAGCATTGTGG | 158 |
|  | nCoV-2019_PL3_42R_158bp | CTGTAATTTGACAGCAGAATTGGC |  |
| 12831-13006 | nCoV-2019_PL3_43F_176bp | AGGATTTGAAATGGGCTAGATTCC | 176 |
|  | nCoV-2019_PL3_43R_176bp | AGCTAAACTACCAAGTACCATACCTC |  |
| 13126-13276 | nCoV-2019_PL3_44F_151bp | GGGACAACCAATCACTAATTGTGT | 151 |
|  | nCoV-2019_PL3_44R_151bp | TGGATGATCTATGTGGCAACGG |  |
| 13431-13572 | nCoV-2019_PL3_45F_142bp | CCATGCTTCAGTCAGCTGATG | 142 |
|  | nCoV-2019_PL3_45R_142bp | ACCAGCTACTTTATCATTGTAGATGTC |  |
| 13723-13903 | nCoV-2019_PL3_46F_181bp | GCTGTTGCTAAACATGACTTCTTTAAG | 181 |
|  | nCoV-2019_PL3_46R_181bp | CATCATCACAACAATTGTATGTGACAAG |  |
| 14035-14188 | nCoV-2019_PL3_47F_154bp | GCTGGTATTGTTGGTGTACTGAC | 154 |
|  | nCoV-2019_PL3_47R_154bp | CCCTGGTCAAGGTTAATATAGGCA |  |
| 14324-14491 | nCoV-2019_PL3_48F_168bp | ACCCAAATTGTGTTAACTGTTTGG | 168 |
|  | nCoV-2019_PL3_48R_168bp | GCTCTCTGAAGTGGTATCCAGT |  |
| 14656-14806 | nCoV-2019_PL3_49F_151bp | GCTTTTCAAACTGTCAAACCCG | 151 |
|  | nCoV-2019_PL3_49R_151bp | AGTAGTCATAATCGCTGATAGCAGC |  |
| 14942-15120 | nCoV-2019_PL3_50F_179bp | CAGCTGGTTTTCCATTTAATAAATGGG | 179 |
|  | nCoV-2019_PL3_50R_179bp | GACACCAGCTACGGTGCGA |  |
| 15289-15450 | nCoV-2019_PL3_51F_162bp | TGGGATTATCCTAAATGTGATAGAGCC | 162 |
|  | nCoV-2019_PL3_51R_162bp | GCCACACATGACCATTTCACTC |  |
| 15597-15766 | nCoV-2019_PL3_52F_170bp | TGTCCGCAATTTACAACACAGAC | 170 |
|  | nCoV-2019_PL3_52R_170bp | CTAGACCTTGAGATGCATAAGTGC |  |
| 15933-16090 | nCoV-2019_PL3_53F_158bp | CCCAGATCCATCAAGAATCCTAGG | 158 |
|  | nCoV-2019_PL3_53R_158bp | ACAAATGAAAGACATCAGCATACTCC |  |
| 16221-16391 | nCoV-2019_PL3_54F_171bp | GCATACAGTCTTACAGGCTGTTGG | 171 |
|  | nCoV-2019_PL3_54R_171bp | GCATTGCAAACATACGGATTAACAG |  |
| 16526-16710 | nCoV-2019_PL3_55F_185bp | GTGTTGGTAGCGATAATGTTACTGAC | 185 |
|  | nCoV-2019_PL3_55R_185bp | CAGCACTTCACGTACAGTAGC |  |
| 16859-17036 | nCoV-2019_PL3_56F_178bp | CTGTTGTTTACCGAGGTACAACAAC | 178 |
|  | nCoV-2019_PL3_56R_178bp | GCAACATTGCTAGAAAACTCATCTG |  |
| 17176-17333 | nCoV-2019_PL3_57F_158bp | GTTGATGCACTATGTGAGAAGGC | 158 |
|  | nCoV-2019_PL3_57R_158bp | GTCTCAGGCAATGCATTTACAGTAC |  |
| 17478-17648 | nCoV-2019_PL3_58F_171bp | GGGCACACTAGAACCAGAATATTTC | 171 |
|  | nCoV-2019_PL3_58R_171bp | CATTGAGCTGATTTGTCTTTATGTGCT |  |
| 17792-17954 | nCoV-2019_PL3_59F_163bp | ATGCTGTAGCCTCAAAGATTTTGG | 163 |
|  | nCoV-2019_PL3_59R_163bp | AGTATGCCTACTTTTGCTCTGGT |  |
| 18089-18265 | nCoV-2019_PL3_60F_177bp | GGTTACATCCTACACAGGCACC | 177 |
|  | nCoV-2019_PL3_60R_177bp | GGGTGATAAACATGTTAGGGTAACC |  |
| 18374-18560 | nCoV-2019_PL3_61F_187bp | CTACAGGTGTTAACCTAGTTGCTG | 187 |
|  | nCoV-2019_PL3_61R_187bp | AGTGTGTCACTTAACATTTGTACAATCT |  |
| 18712-18860 | nCoV-2019_PL3_62F_149bp | GCCTGTTGGCATCATTCTATTGG | 149 |
|  | nCoV-2019_PL3_62R_149bp | GCATCACAACTAGCTACATGTGC |  |
| 19019-19180 | nCoV-2019_PL3_63F_162bp | CAGTTCTTCACGACATTGGTAACC | 162 |
|  | nCoV-2019_PL3_63R_162bp | CACCATCTGTGAATTTGTCAGAATG |  |
| 19321-19496 | nCoV-2019_PL3_64F_176bp | ACACCAGCTTTTGATAAAAGTGCT | 176 |
|  | nCoV-2019_PL3_64R_176bp | TGTCTACAGACAGCACCACC |  |
| 19672-19835 | nCoV-2019_PL3_65F_164bp | CAACAGGGTGAAGTACCAGTTTC | 164 |
|  | nCoV-2019_PL3_65R_164bp | AGTATTTTCACCTCTGGTACTGGT |  |
| 19962-20135 | nCoV-2019_PL3_66F_174bp | GATTTGTGCACCACTCACTGTC | 174 |
|  | nCoV-2019_PL3_66R_174bp | ACGGCTTCTCCAATTAATGTGAC |  |
| 20291-20474 | nCoV-2019_PL3_67F_184bp | GGTATAAATTAGAAGGCTATGCCTTCG | 184 |
|  | nCoV-2019_PL3_67R_184bp | GTTTGCGCATCTGTTATGAAATAGT |  |
| 20593-20774 | nCoV-2019_PL3_68F_182bp | ACAGAAATTTCATTTATGCTTTGGTG | 182 |
|  | nCoV-2019_PL3_68R_182bp | CCTTTAGGTAATGTTGCACTATCACC |  |
| 20910-21066 | nCoV-2019_PL3_69F_157bp | TTTAAGACAGTGGTTGCCTACGG | 157 |
|  | nCoV-2019_PL3_69R_157bp | AGTCTTAGGGTCGTACATATCACTAAT |  |
| 21201-21339 | nCoV-2019_PL3_70F_139bp | TAAGCTCATGGGACACTTCGC | 139 |
|  | nCoV-2019_PL3_70R_139bp | ATTTGCATGCATGACATAACCATC |  |
| 21428-21624 | nCoV-2019_PL3_71F_197bp | CTGCTGTTATGTCTTTAAAAGAAGGTCA | 197 |
|  | nCoV-2019_PL3_71R_197bp | CTGGTTGTAAGATTAACACACTGACT |  |
| 21775-21930 | nCoV-2019_PL3_72F_156bp | TGGGACCAATGGTACTAAGAGGT | 156 |
|  | nCoV-2019_PL3_72R_156bp | GCGTTATTAACAATAAGTAGGGACTGG |  |
| 22077-22240 | nCoV-2019_PL3_73F_164bp | CTCAGCCTTTTCTTATGGACCTTG | 164 |
|  | nCoV-2019_PL3_73R_164bp | CAATGGTTCTAAAGCCGAAAAACC |  |
| 22363-22537 | nCoV-2019_PL3_74F_175bp | GGGTTATCTTCAACCTAGGACTTTTC | 175 |
|  | nCoV-2019_PL3_74R_175bp | AGATTCTGTTGGTTGGACTCTAAAGT |  |
| 22638-22804 | nCoV-2019_PL3_75F_167bp | GCAACTGTGTTGCTGATTATTCTG | 167 |
|  | nCoV-2019_PL3_75R_167bp | TTGCCCTGGAGCGATTTGTC |  |
| 22893-23093 | nCoV-2019_PL3_76F_201bp | AGGTTGGTGGTAATTATAATTACCTGT | 201 |
|  | nCoV-2019_PL3_76R_201bp | CTACTCTGTATGGTTGGTAACCAACA |  |
| 23240-23426 | nCoV-2019_PL3_77F_187bp | CTGCCTTTCCAACAATTTGGCA | 187 |
|  | nCoV-2019_PL3_77R_187bp | CAGGGACTTCTGTGCAGTTAACA |  |
| 23555-23749 | nCoV-2019_PL3_78F_195bp | CCCATTGGTGCAGGTATATGCG | 195 |
|  | nCoV-2019_PL3_78R_195bp | CACTGGTAGAATTTCTGTGGTAACAC |  |
| 23893-24057 | nCoV-2019_PL3_79F_165bp | CACCCAAGAAGTTTTTGCACAAG | 165 |
|  | nCoV-2019_PL3_79R_165bp | CCAGCATCTGCAAGTGTCAC |  |
| 24197-24358 | nCoV-2019_PL3_80F_162bp | GCGGGTACAATCACTTCTGGT | 162 |
|  | nCoV-2019_PL3_80R_162bp | GCCAATAGCACTATTAAATTGGTTGG |  |
| 24509-24673 | nCoV-2019_PL3_81F_165bp | CGTCTTGACAAAGTTGAGGCTG | 165 |
|  | nCoV-2019_PL3_81R_165bp | TGATTGTCCAAGTACACACTCTG |  |
| 24820-24986 | nCoV-2019_PL3_82F_167bp | AGCACACTTTCCTCGTGAAGG | 167 |
|  | nCoV-2019_PL3_82R_167bp | GCAAAGGATCATAAACTGTGTTGTTG |  |
| 25071-25247 | nCoV-2019_PL3_83F_177bp | CTGGCATTAATGCTTCAGTTGTAAAC | 177 |
|  | nCoV-2019_PL3_83R_177bp | TTACTATGGCAATCAAGCCAGC |  |
| 25348-25553 | nCoV-2019_PL3_84F_206bp | GCCAGTGCTCAAAGGAGTCAAAT | 206 |
|  | nCoV-2019_PL3_84R_206bp | GCAAGAAGTGCAACGCCAAC |  |
| 25720-25917 | nCoV-2019_PL3_85F_198bp | GCTTTAGTCTACTTCTTGCAGAGT | 198 |
|  | nCoV-2019_PL3_85R_198bp | TGTGCCATCACCTGAAGTAATGAC |  |
| 26018-26205 | nCoV-2019_PL3_86F_188bp | CAGACTATTACCAGCTGTACTCAACTCA | 188 |
|  | nCoV-2019_PL3_86R_188bp | AGTAGTCGTCGTCGGTTCATCAT |  |
| 26316-26476 | nCoV-2019_PL3_87F_161bp | GGTATTCTTGCTAGTTACACTAGCCA | 161 |
|  | nCoV-2019_PL3_87R_161bp | TCGTTTAGACCAGAAGATCAGGAAC |  |
| 26634-26800 | nCoV-2019_PL3_88F_167bp | GCCTATGCCAACAGGAATAGGT | 167 |
|  | nCoV-2019_PL3_88R_167bp | AGCCACATCAAGCCTACAAGAC |  |
| 26958-27122 | nCoV-2019_PL3_89F_165bp | CGTGGACATCTTCGTATTGCTG | 165 |
|  | nCoV-2019_PL3_89R_165bp | CCTGTAGCGACTGTATGCAGC |  |
| 27258-27431 | nCoV-2019_PL3_90F_174bp | GAGGACTTTTAAAGTTTCCATTTGGAA | 174 |
|  | nCoV-2019_PL3_90R_174bp | GCGAGTGTTATCAGTGCCAAG |  |
| 27561-27752 | nCoV-2019_PL3_91F_192bp | GACTTGCTTTAGCACTCAATTTGCT | 192 |
|  | nCoV-2019_PL3_91R_192bp | GTCTTTCTTTTGAGTGTGAAGCAAAGT |  |
| 27879-28060 | nCoV-2019_PL3_92F_182bp | CACGCCTAAACGAACATGAAATT | 182 |
|  | nCoV-2019_PL3_92R_182bp | GGTGCTGATTTTCTAGCTCCTACTC |  |
| 28178-28338 | nCoV-2019_PL3_93F_161bp | GGGTAGTCTTGTAGTGCGTTGT | 161 |
|  | nCoV-2019_PL3_93R_161bp | TCTGAGGGTCCACCAAACGT |  |
| 28506-28659 | nCoV-2019_PL3_94F_154bp | GCAGTCCAGATGACCAAATTGG | 154 |
|  | nCoV-2019_PL3_94R_154bp | CCGTCTTTGTTAGCACCATAGGG |  |
| 28785-28941 | nCoV-2019_PL3_95F_157bp | TCTACGCAGAAGGGAGCAGAGG | 157 |
|  | nCoV-2019_PL3_95R_157bp | AGCAGCAAAGCAAGAGCAGC |  |
| 29104-29261 | nCoV-2019_PL3_96F_158bp | TGGTCCAGAACAAACCCAAGGA | 158 |
|  | nCoV-2019_PL3_96R_158bp | ACGTTCCCGAAGGTGTGACT |  |
| 29410-29563 | nCoV-2019_PL3_97F_154bp | TCAAGCCTTACCGCAGAGAC | 154 |
|  | nCoV-2019_PL3_97R_154bp | GCCCATCTGCCTTGTGTGGT |  |
| 29625-29770 | nCoV-2019_PL3_98F_146bp | CTCGTAACTACATAGCACAAGTAGATG | 146 |
|  | nCoV-2019_PL3_98R_146bp | TGTTCACTGTACACTCGATCGT |  |
