# Supplementary material for: Identification and Analysis of SARS-CoV-2 Alpha Variants in the Largest Taiwan COVID-19 Outbreak in 2021
Source: Front Med (Lausanne). 2022 Apr 25;9:869818. doi: 10.3389/fmed.2022.869818 (PMC9081839; doi:10.3389/fmed.2022.869818)
Supplement: Supplementary file 2 [file Table_2.DOCX]

**SUPPLEMENTARY TABLE 2 |** The full list of single-nucleotide variants and insertion-deletion analysis of the KMUH-3 to KMUH-7 genomes compared to the reference Wuhan-Hu-1/2019.

**KMUH-3**

| **Nucleotide Position** | **Gene** | **Protein** | **Reference** | **Variant** | **SNV Frequency** | **InDel Frequency** | **Codon change** | **Variant type** |
| --- | --- | --- | --- | --- | --- | --- | --- | --- |
|  |  |  |  |  |  |  |  |  |
| 241 | --- | --- | C | T | 100% | --- | --- | --- |
| 913 | ORF1ab | NSP2 | C | T | 100% | --- | S216S | synonymous |
| 1292 | ORF1ab | NSP2 | T | C | 100% | --- | F343L | missense |
| 3037 | ORF1ab | NSP3 | C | T | 100% | --- | F924F | synonymous |
| 3267 | ORF1ab | NSP3 | C | T | 100% | --- | T1001I | missense |
| 3866 | ORF1ab | NSP3 | C | A | 26.20% | --- | Q1201K | missense |
| 5388 | ORF1ab | NSP3 | C | A | 100% | --- | A1708D | missense |
| 5986 | ORF1ab | NSP3 | C | T | 100% | --- | F1907F | synonymous |
| 6954 | ORF1ab | NSP3 | T | C | 100% | --- | I2230T | missense |
| 10504 | ORF1ab | NSP5 | TTA | --- | --- | 15.10% | 3413-3414del | deletion |
| 10533 | ORF1ab | NSP5 | G | T | 5.60% | --- | C3423F | missense |
| 11288 | ORF1ab | NSP6 | TCTGGTTTT | --- | --- | 100% | 3675-3677del | deletion |
| 11355 | ORF1ab | NSP6 | C | T | 5.20% | --- | R3697V | missense |
| 11454 | ORF1ab | NSP6 | C | T | 14.50% | --- | A3730V | missense |
| 11750 | ORF1ab | NSP6 | C | T | 6.50% | --- | L3829F | missense |
| 11873 | ORF1ab | NSP7 | G | A | 5.20% | --- | V3870I | missense |
| 13541 | ORF1ab | NSP12 | C | G | 5.30% | --- | A4426G | synonymous |
| 14408 | ORF1ab | NSP12 | C | T | 100% | --- | P314L | missense |
| 14676 | ORF1ab | NSP12 | C | T | 100% | --- | P4804P | synonymous |
| 15279 | ORF1ab | NSP12 | C | T | 100% | --- | H5005H | synonymous |
| 16176 | ORF1ab | NSP12 | T | C | 100% | --- | T5304T | synonymous |
| 19186 | ORF1ab | NSP14 | C | T | 100% | --- | L6308L | synonymous |
| 19961 | ORF1ab | NSP15 | C | T | 100% | --- | T2165M | missense |
| 21077 | ORF1ab | NSP16 | C | T | 5.90% | --- | T6938I | missense |
| 21766 | S | Spike | A | C | 100% | --- | I68I | synonymous |
| 21767 | S | Spike | CATGTC | --- | --- | 100% | 69-70del | deletion |
| 21992 | S | Spike | TAT | --- | --- | 100% | 144del | deletion |
| 22013 | S | Spike | A | C | 9.00% | --- | S151R | missense |
| 23063 | S | Spike | A | T | 100% | --- | N501Y | missense |
| 23271 | S | Spike | C | A | 100% | --- | A570D | missense |
| 23403 | S | Spike | A | G | 100% | --- | D614G | missense |
| 23525 | S | Spike | C | T | 6.00% | --- | H655Y | missense |
| 23604 | S | Spike | C | A | 100% | --- | P681H | missense |
| 23709 | S | Spike | C | T | 100% | --- | T716I | missense |
| 24506 | S | Spike | T | G | 100% | --- | S982A | missense |
| 24914 | S | Spike | G | C | 100% | --- | D1118H | missense |
| 27215 | ORF6 | NS6 | T | G | 5.70% | --- | V5G | missense |
| 27972 | ORF8 | NS8 | C | T | 100% | --- | Q27* | missense |
| 28048 | ORF8 | NS8 | G | T | 100% | --- | R52I | missense |
| 28111 | ORF8 | NS8 | A | G | 100% | --- | Y73C | missense |
| 28272 | --- | --- | A | --- | --- | 100% | --- | deletion |
| 28280 | N | N | G | C | 100% | --- | D3L | missense |
| 28281 | N | N | A | T |  |  |  |  |
| 28282 | N | N | T | A |  |  |  |  |
| 28881 | N | N | G | A | 100% | --- | R203K | missense |
| 28882 | N | N | G | A |  |  |  |  |
| 28883 | N | N | G | C | 100% | --- | G204R | missense |
| 28977 | N | N | C | T | 100% | --- | S235F | missense |

**KMUH-4**

| **Nucleotide Position** | **Gene** | **Protein** | **Reference** | **Variant** | **SNV Frequency** | **InDel Frequency** | **Codon change** | **Variant type** |
| --- | --- | --- | --- | --- | --- | --- | --- | --- |
|  |  |  |  |  |  |  |  |  |
| 241 | --- | --- | C | T | 100.00% | --- | --- | --- |
| 913 | ORF1ab | NSP2 | C | T | 100.00% | --- | S216S | synonymous |
| 3037 | ORF1ab | NSP3 | C | T | 100.00% | --- | F924F | synonymous |
| 3267 | ORF1ab | NSP3 | C | T | 100.00% | --- | T1001I | missense |
| 5144 | ORF1ab | NSP3 | C | T | 100.00% | --- | L1627L | synonymous |
| 5388 | ORF1ab | NSP3 | C | A | 100.00% | --- | A1708D | missense |
| 5812 | ORF1ab | NSP3 | C | T | 100.00% | --- | D1849D | synonymous |
| 5986 | ORF1ab | NSP3 | C | T | 100.00% | --- | F1907F | synonymous |
| 6954 | ORF1ab | NSP3 | T | C | 100.00% | --- | I2230T | missense |
| 11288 | ORF1ab | NSP6 | TCTGGTTTT | --- | --- | 100.00% | 3675-3677del | deletion |
| 12253 | ORF1ab | NSP8 | C | T | 100.00% | --- | A3996A | synonymous |
| 14408 | ORF1ab | NSP12 | C | T | 100.00% | --- | P314L | missense |
| 14676 | ORF1ab | NSP12 | C | T | 100.00% | --- | P4804P | synonymous |
| 15279 | ORF1ab | NSP12 | C | T | 100.00% | --- | H5005H | synonymous |
| 15895 | ORF1ab | NSP12 | C | T | 100.00% | --- | L5211L | synonymous |
| 16176 | ORF1ab | NSP12 | T | C | 100.00% | --- | T5304T | synonymous |
| 21766 | S | Spike | A | C | 100.00% | --- | I68I | synonymous |
| 21767 | S | Spike | CATGTC | --- | --- | 100.00% | 69-70del | deletion |
| 21992 | S | Spike | TAT | --- | --- | 100.00% | 144del | deletion |
| 23063 | S | Spike | A | T | 100.00% | --- | N501Y | missense |
| 23271 | S | Spike | C | A | 100.00% | --- | A570D | missense |
| 23403 | S | Spike | A | G | 100.00% | --- | D614G | missense |
| 23604 | S | Spike | C | A | 100.00% | --- | P681H | missense |
| 23709 | S | Spike | C | T | 100.00% | --- | T716I | missense |
| 24506 | S | Spike | T | G | 100.00% | --- | S982A | missense |
| 24914 | S | Spike | G | C | 100.00% | --- | D1118H | missense |
| 24919 | S | Spike | C | T | 100.00% | --- | N1119N | synonymous |
| 25273 | S | Spike | G | C | 100.00% | --- | M1237I | missense |
| 27513 | ORF7a | NS7a | C | T | 100.00% | --- | Y40Y | synonymous |
| 27869 | ORF8 | NS8 | T | C | 100.00% | --- | N38N | synonymous |
| 27972 | ORF8 | NS8 | C | T | 100.00% | --- | Q27* | missense |
| 28048 | ORF8 | NS8 | G | T | 100.00% | --- | R52I | missense |
| 28111 | ORF8 | NS8 | A | G | 100.00% | --- | Y73C | missense |
| 28271 | --- | --- | A | --- | --- | 100.00% | --- | deletion |
| 28280 | N | N | G | C | 100.00% | --- | D3L | missense |
| 28281 | N | N | A | T | 100.00% | --- |  |  |
| 28282 | N | N | T | A | 100.00% | --- |  |  |
| 28881 | N | N | G | A | 100.00% | --- | R203K | missense |
| 28882 | N | N | G | A | 100.00% | --- |  |  |
| 28883 | N | N | G | C | 100.00% | --- | G204R | missense |
| 28957 | N | N | C | T | 100.00% | --- | N228N | synonymous |
| 28977 | N | N | C | T | 100.00% | --- | S235F | missense |

**KMUH-5**

| **Nucleotide Position** | **Gene** | **Protein** | **Reference** | **Variant** | **SNV Frequency** | **InDel Frequency** | **Codon change** | **Variant type** |
| --- | --- | --- | --- | --- | --- | --- | --- | --- |
|  |  |  |  |  |  |  |  |  |
| 241 | --- | --- | C | T | 100% | --- | --- | --- |
| 913 | ORF1ab | NSP2 | C | T | 100% | --- | S216S | synonymous |
| 2459 | ORF1ab | NSP2 | C | T | 100% | --- | P732S | missense |
| 3037 | ORF1ab | NSP3 | C | T | 100% | --- | F924F | synonymous |
| 3267 | ORF1ab | NSP3 | C | T | 100% | --- | T1001I | missense |
| 5144 | ORF1ab | NSP3 | C | T | 100% | --- | L1627L | synonymous |
| 5388 | ORF1ab | NSP3 | C | A | 100% | --- | A1708D | missense |
| 5812 | ORF1ab | NSP3 | C | T | 100% | --- | D1849D | synonymous |
| 5986 | ORF1ab | NSP3 | C | T | 100% | --- | F1907F | synonymous |
| 6954 | ORF1ab | NSP3 | T | C | 100% | --- | I2230T | missense |
| 11288 | ORF1ab | NSP6 | TCTGGTTTT | --- | --- | 100% | 3675-3677del | deletion |
| 12253 | ORF1ab | NSP8 | C | T | 100% | --- | A3996A | synonymous |
| 14408 | ORF1ab | NSP12 | C | T | 100% | --- | P314L | missense |
| 14676 | ORF1ab | NSP12 | C | T | 100% | --- | P4804P | synonymous |
| 15279 | ORF1ab | NSP12 | C | T | 100% | --- | H5005H | synonymous |
| 15895 | ORF1ab | NSP12 | C | T | 100% | --- | L5211L | synonymous |
| 16176 | ORF1ab | NSP12 | T | C | 100% | --- | T5304T | synonymous |
| 21766 | S | Spike | A | C | 100% | --- | I68I | synonymous |
| 21767 | S | Spike | CATGTC | --- | --- | 100% | 69-70del | deletion |
| 21992 | S | Spike | TAT | --- | --- | 100% | 144del | deletion |
| 23063 | S | Spike | A | T | 100% | --- | N501Y | missense |
| 23271 | S | Spike | C | A | 100% | --- | A570D | missense |
| 23403 | S | Spike | A | G | 100% | --- | D614G | missense |
| 23604 | S | Spike | C | A | 100% | --- | P681H | missense |
| 23709 | S | Spike | C | T | 100% | --- | T716I | missense |
| 24506 | S | Spike | T | G | 100% | --- | S982A | missense |
| 24914 | S | Spike | G | C | 100% | --- | D1118H | missense |
| 24919 | S | Spike | C | T | 100% | --- | N1119N | synonymous |
| 25273 | S | Spike | G | C | 100% | --- | M1237I | missense |
| 27869 | ORF7b | NS7b | T | C | 100% | --- | N38N | synonymous |
| 28048 | ORF8 | NS8 | G | T | 100% | --- | R52I | missense |
| 28111 | ORF8 | NS8 | A | G | 100% | --- | Y73C | missense |
| 28272 | --- | --- | A | --- | --- | 100% | --- | deletion |
| 28280 | N | N | G | C | 100% | --- | D3L | missense |
| 28281 | N | N | A | T | 100% | --- |  |  |
| 28282 | N | N | T | A | 100% | --- |  |  |
| 28881 | N | N | G | A | 100% | --- | R203K | missense |
| 28882 | N | N | G | A | 100% | --- |  |  |
| 28883 | N | N | G | C | 100% | --- | G204R | missense |
| 28957 | N | N | C | T | 100% | --- | N228N | synonymous |
| 28977 | N | N | C | T | 100% | --- | S235F | missense |

**KMUH-6**

| **Nucleotide Position** | **Gene** | **Protein** | **Reference** | **Variant** | **SNV Frequency** | **InDel Frequency** | **Codon change** | **Variant type** |
| --- | --- | --- | --- | --- | --- | --- | --- | --- |
|  |  |  |  |  |  |  |  |  |
| 241 | --- | --- | C | T | 100% | --- | --- | --- |
| 913 | ORF1ab | NSP2 | C | T | 100% | --- | S216S | synonymous |
| 3037 | ORF1ab | NSP3 | C | T | 100% | --- | F924F | synonymous |
| 3267 | ORF1ab | NSP3 | C | T | 100% | --- | T1001I | missense |
| 5144 | ORF1ab | NSP3 | C | T | 100% | --- | L1627L | synonymous |
| 5388 | ORF1ab | NSP3 | C | A | 100% | --- | A1708D | missense |
| 5812 | ORF1ab | NSP3 | C | T | 100% | --- | D1849D | synonymous |
| 5986 | ORF1ab | NSP3 | C | T | 100% | --- | F1907F | synonymous |
| 6954 | ORF1ab | NSP3 | T | C | 100% | --- | I2230T | missense |
| 11288 | ORF1ab | NSP6 | TCTGGTTTT | --- | --- | 100% | 3675-3677del | deletion |
| 12253 | ORF1ab | NSP6 | C | T | 100% | --- | A3996A | synonymous |
| 13541 | ORF1ab | NSP6 | C | G | 8.50% | --- | A4426G | missense |
| 14408 | ORF1ab | NSP12 | C | T | 100% | --- | P314L | missense |
| 14676 | ORF1ab | NSP12 | C | T | 100% | --- | P4804P | synonymous |
| 15279 | ORF1ab | NSP12 | C | T | 100% | --- | H5005H | synonymous |
| 15895 | ORF1ab | NSP12 | C | T | 100% | --- | L5211L | synonymous |
| 16176 | ORF1ab | NSP12 | T | C | 100% | --- | T5304T | synonymous |
| 20233 | ORF1ab | NSP15 | C | T | 100% | --- | P2256S | missense |
| 21766 | S | Spike | A | C | 100% | --- | I68I | synonymous |
| 21767 | S | Spike | CATGTC | --- | --- | 100% | 69-70del | deletion |
| 21992 | S | Spike | TAT | --- | --- | 100% | 144del | deletion |
| 23063 | S | Spike | A | T | 100% | --- | N501Y | missense |
| 23271 | S | Spike | C | A | 100% | --- | A570D | missense |
| 23403 | S | Spike | A | G | 100% | --- | D614G | missense |
| 23604 | S | Spike | C | A | 100% | --- | P681H | missense |
| 23607 | S | Spike | G | T | 47.70% | --- | R682L | missense |
| 23709 | S | Spike | C | T | 100% | --- | T716I | missense |
| 24506 | S | Spike | T | G | 100% | --- | S982A | missense |
| 24914 | S | Spike | G | C | 100% | --- | D1118H | missense |
| 24919 | S | Spike | C | T | 100% | --- | N1119N | synonymous |
| 25273 | S | Spike | G | C | 100% | --- | M1237I | missense |
| 27513 | ORF7a | NS7a | C | T | 100% | --- | Y40Y | synonymous |
| 27869 | ORF8 | NS7b | T | C | 100% | --- | N38N | synonymous |
| 27972 | ORF8 | NS8 | C | T | 100% | --- | Q27* | missense |
| 28048 | ORF8 | NS8 | G | T | 100% | --- | R52I | missense |
| 28111 | ORF8 | NS8 | A | G | 100% | --- | Y73C | missense |
| 28272 | --- | --- | A | --- | --- | 100% | --- | deletion |
| 28280 | N | N | G | C | 100% | --- | D3L | missense |
| 28281 | N | N | A | T |  |  |  |  |
| 28282 | N | N | T | A |  |  |  |  |
| 28881 | N | N | G | A | 100% | --- | R203K | missense |
| 28882 | N | N | G | A |  |  |  |  |
| 28883 | N | N | G | C | 100% | --- | G204R | missense |
| 28957 | N | N | C | T | 100% | --- | N228N | synonymous |
| 28977 | N | N | C | T | 100% | --- | S235F | missense |
| 29137 | N | N | C | T | 100% | --- | D288D | synonymous |
| 29871 | --- | --- | A | G | 30.50% | --- | --- | --- |
| 29873 | --- | --- | A | G | 24.70% | --- | --- | --- |

**KMUH-7**

| **Nucleotide Position** | **Gene** | **Protein** | **Reference** | **Variant** | **SNV Frequency** | **InDel Frequency** | **Codon change** | **Variant type** |
| --- | --- | --- | --- | --- | --- | --- | --- | --- |
|  |  |  |  |  |  |  |  |  |
| 241 | --- | --- | C | T | 100.00% | --- | --- | --- |
| 913 | ORF1ab | NSP2 | C | T | 100.00% | --- | S216S | synonymous |
| 2911 | ORF1ab | NSP3 | T | G | 5.10% | --- | T882T | synonymous |
| 3037 | ORF1ab | NSP3 | C | T | 100.00% | --- | F924F | synonymous |
| 3267 | ORF1ab | NSP3 | C | T | 100.00% | --- | T1001I | missense |
| 4456 | ORF1ab | NSP3 | C | T | 5.70% | --- | A1397A | synonymous |
| 5144 | ORF1ab | NSP3 | C | T | 100.00% | --- | L1627L | synonymous |
| 5388 | ORF1ab | NSP3 | C | A | 100.00% | --- | A1708D | missense |
| 5812 | ORF1ab | NSP3 | C | T | 100.00% | --- | D1849D | synonymous |
| 5986 | ORF1ab | NSP3 | C | T | 100.00% | --- | F1907F | synonymous |
| 6825 | ORF1ab | NSP3 | A | C | 5.30% | --- | N2187T | missense |
| 6954 | ORF1ab | NSP3 | T | C | 100.00% | --- | I2230T | missense |
| 10159 | ORF1ab | NSP5 | A | G | 100.00% | --- | V3298V | synonymous |
| 10533 | ORF1ab | NSP5 | G | T | 33.30% | --- | C3423F | missense |
| 11288 | ORF1ab | NSP6 | TCTGGTTTT | --- | --- | 100.00% | 3675-3677del | deletion |
| 11401 | ORF1ab | NSP6 | G | A | 5.40% | --- | M3712I | missense |
| 11522 | ORF1ab | NSP6 | T | G | 11.10% | --- | F3753V | missense |
| 11750 | ORF1ab | NSP6 | C | T | 9.00% | --- | L3829F | missense |
| 12253 | ORF1ab | NSP8 | C | T | 100.00% | --- | A3996A | synonymous |
| 14408 | ORF1ab | NSP12 | C | T | 100.00% | --- | P314L | missense |
| 14676 | ORF1ab | NSP12 | C | T | 100.00% | --- | P4804P | synonymous |
| 15279 | ORF1ab | NSP12 | C | T | 100.00% | --- | H5005H | synonymous |
| 15895 | ORF1ab | NSP12 | C | T | 100.00% | --- | L5211L | synonymous |
| 16176 | ORF1ab | NSP12 | T | C | 100.00% | --- | T5304T | synonymous |
| 20486 | ORF1ab | NSP15 | A | C | 16.90% | --- | K6741T | missense |
| 21766 | S | Spike | A | C | 100% | --- | I68I | synonymous |
| 21767 | S | Spike | CATGTC | --- | --- | 100% | 69-70del | deletion |
| 21992 | S | Spike | TAT | --- | --- | 100.00% | 144del | deletion |
| 23063 | S | Spike | A | T | 100.00% | --- | N501Y | missense |
| 23271 | S | Spike | C | A | 100.00% | --- | A570D | missense |
| 23403 | S | Spike | A | G | 100.00% | --- | D614G | missense |
| 23604 | S | Spike | C | A | 100.00% | --- | P681H | missense |
| 23709 | S | Spike | C | T | 100.00% | --- | T716I | missense |
| 24506 | S | Spike | T | G | 100.00% | --- | S982A | missense |
| 24914 | S | Spike | G | C | 100.00% | --- | D1118H | missense |
| 24919 | S | Spike | C | T | 100.00% | --- | N1119N | synonymous |
| 25273 | S | Spike | G | C | 100.00% | --- | M1237I | missense |
| 26110 | ORF3a | NS3 | C | T | 18.10% | --- | P240S | missense |
| 27513 | ORF7a | NS7a | C | T | 100.00% | --- | Y40Y | synonymous |
| 27661 | ORF7a | NS7a | C | T | 100.00% | --- | Q90* | missense |
| 27869 | ORF8 | NS8 | T | C | 100.00% | --- | N38N | synonymous |
| 27972 | ORF8 | NS8 | C | T | 100.00% | --- | Q27* | missense |
| 28048 | ORF8 | NS8 | G | T | 100.00% | --- | R52I | missense |
| 28111 | ORF8 | NS8 | A | G | 100.00% | --- | Y73C | missense |
| 28272 | --- | --- | A | --- | --- | 100.00% | --- | deletion |
| 28280 | N | N | G | C | 100.00% | --- | D3L | missense |
| 28281 | N | N | A | T | 100.00% | --- |  |  |
| 28282 | N | N | T | A | 100.00% | --- |  |  |
| 28340 | N | N | T | A | 8.70% | --- | S23T | missense |
| 28881 | N | N | G | A | 100.00% | --- | R203K | missense |
| 28882 | N | N | G | A | 100.00% | --- |  |  |
| 28883 | N | N | G | C | 100.00% | --- | G204R | missense |
| 28957 | N | N | C | T | 100.00% | --- | N228N | synonymous |
| 28977 | N | N | C | T | 100.00% | --- | S235F | missense |
