# Supplementary material for: Identification and Analysis of SARS-CoV-2 Alpha Variants in the Largest Taiwan COVID-19 Outbreak in 2021
Source: Front Med (Lausanne). 2022 Apr 25;9:869818. doi: 10.3389/fmed.2022.869818 (PMC9081839; doi:10.3389/fmed.2022.869818)
Supplement: Supplementary file 3 [file Table_3.DOCX]

**SUPPLEMENTARY TABLE 3 |** Clade replacement over time between January 2020 and December 2021 in Taiwan.^a^

|  | 2020 | | | | | | | | | | | | 2021 | | | | | | | | | | | |
| --- | --- | --- | --- | --- | --- | --- | --- | --- | --- | --- | --- | --- | --- | --- | --- | --- | --- | --- | --- | --- | --- | --- | --- | --- |
| Clade | Jan | Feb | Mar | Apr | May | Jun | Jul | Aug | Sep | Oct | Nov | Dec | Jan | Feb | Mar | Apr | May | Jun | Jul | Aug | Sep | Oct | Nov | Dec |
| 19A | 5 | 11 | 33 | 5 | - | - | - | - | - | 1 | 1 | - | - | - | - | - | - | - | - | - | - | - | - | - |
| 19B | 2 | - | 7 | 2 | - | - | - | - | - | - | - | - | - | - | - | - | - | - | - | - | - | - | - | - |
| 20A | - | - | 26 | 2 | - | - | - | - | 1 | 1 | 6 | 7 | 1 | - | - | 1 | - | - | - | - | - | - | - | - |
| 20B | - | - | 17 | - | - | - | 1 | 1 | - | 1 | 5 | 2 | - | - | 1 | - | - | - | - | - | - | - | - | - |
| 20C | - | - | 17 | 1 | - | - | - | - | - | - | 2 | - | 1 | - | - | - | - | - | - | - | - | - | - | - |
| 20E (EU1) | - | - | - | - | - | - | - | - | 1 | - | - | - | - | 2 | - | - | - | - | - | - | - | - | - | - |
| 20G | - | - | - | - | - | - | - | - | - | 1 | - | 4 | 4 | - | - | - | - | - | - | - | - | - | - | - |
| 20H (Beta, V2) | - | - | - | - | - | - | - | - | - | - | - | - | - | - | 2 | 3 | - | - | - | - | - | - | - | - |
| 20I (Alpha, V1) | - | - | - | - | - | - | - | - | - | - | - | 3 | 2 | - | 1 | 9 | 23 | 20 | 2 | - | - | - | - | - |
| 20J (Gamma, V3) | - | - | - | - | - | - | - | - | - | - | - | - | 5 | - | - | - | - | - | 1 | - | - | - | - | - |
| 21A (Delta) | - | - | - | - | - | - | - | - | - | - | - | - | - | - | - | - | - | - | 1 | 1 | - | - | - | - |
| 21C (Epsilon) | - | - | - | - | - | - | - | - | - | - | - | - | 8 | - | - | - | - | - | - | - | - | - | - | - |
| 21I (Delta) | - | - | - | - | - | - | - | - | - | - | - | - | - | - | - | - | 1 | - | 2 | 1 |  | - | - | - |
| 21J (Delta) | - | - | - | - | - | - | - | - | - | - | - | - | - | - | - | - | - | 3 | 3 | 2 | 1 | - | - | - |

^a^ The data were retrieved from the GISAID on the January 2, 2022.
