# Supplementary figures and images for: Identification and Analysis of SARS-CoV-2 Alpha Variants in the Largest Taiwan COVID-19 Outbreak in 2021
Source: Front Med (Lausanne). 2022 Apr 25;9:869818. doi: 10.3389/fmed.2022.869818 (PMC9081839; doi:10.3389/fmed.2022.869818)

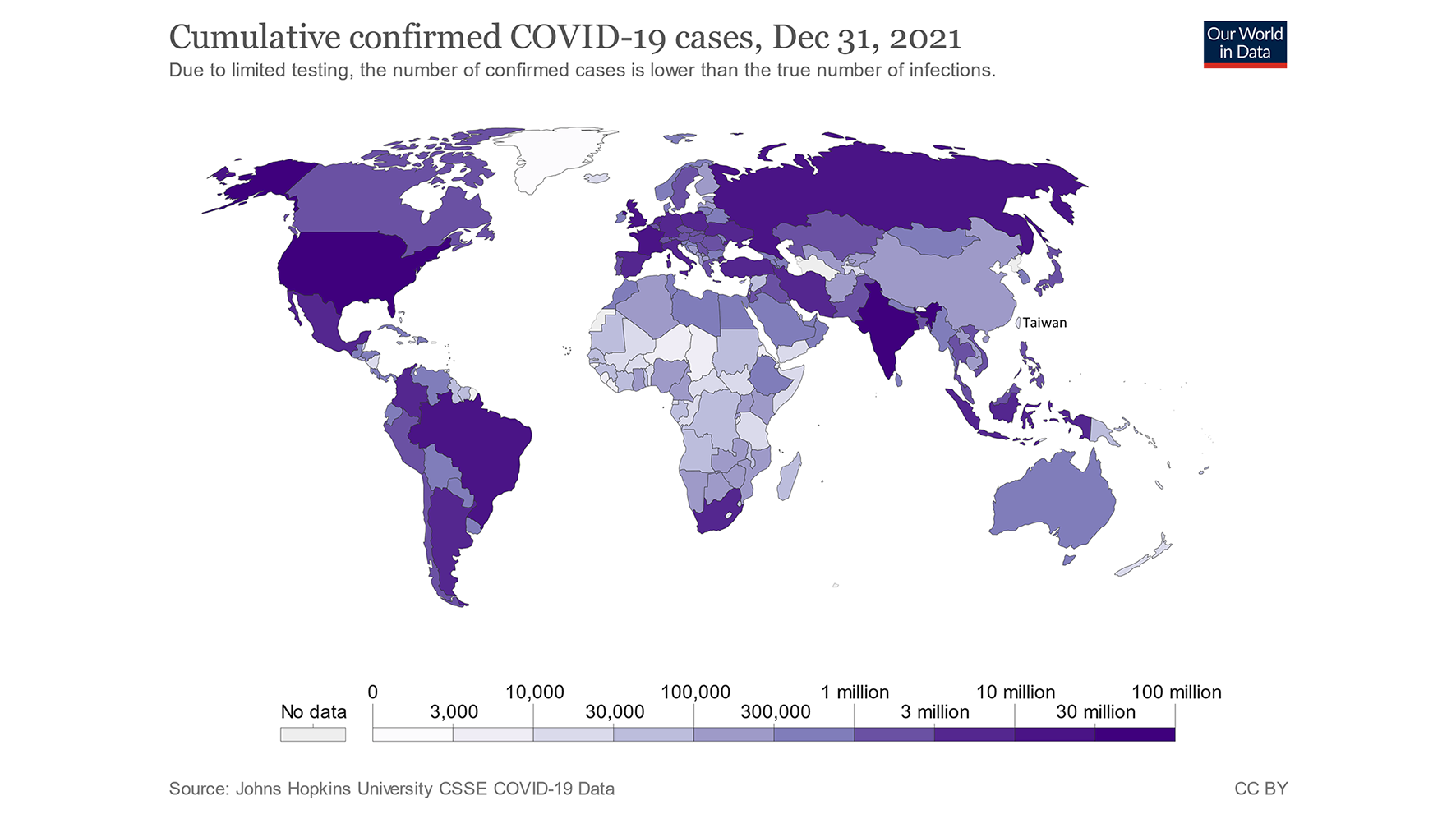

Supplement: Supplementary Figure 1 — Cumulative confirmed COVID-19 cases from 22 January 2020 to 31 December 2021. Hannah Ritchie, Edouard Mathieu, Lucas Rodés-Guirao, Cameron Appel, Charlie Giattino, Esteban Ortiz-Ospina, Joe Hasell, Bobbie Macdonald, Diana Beltekian, and Max Roser (2020) – “Coronavirus Pandemic (COVID-19).” Published online at OurWorldInData.org. Retrieved from: “https://ourworldindata.org/explorers/coronavirus-data-explorer?tab=map&zoom ToSelection=true&time=2021-12-31&facet=none&hideControls=true&Metric= Confirmed+cases&Interval=Cumulative&Relative+to+Population=false&Color+ by+test+positivity=false&country=∼OWID_WRL” (online resource). [file Image_1.TIF]

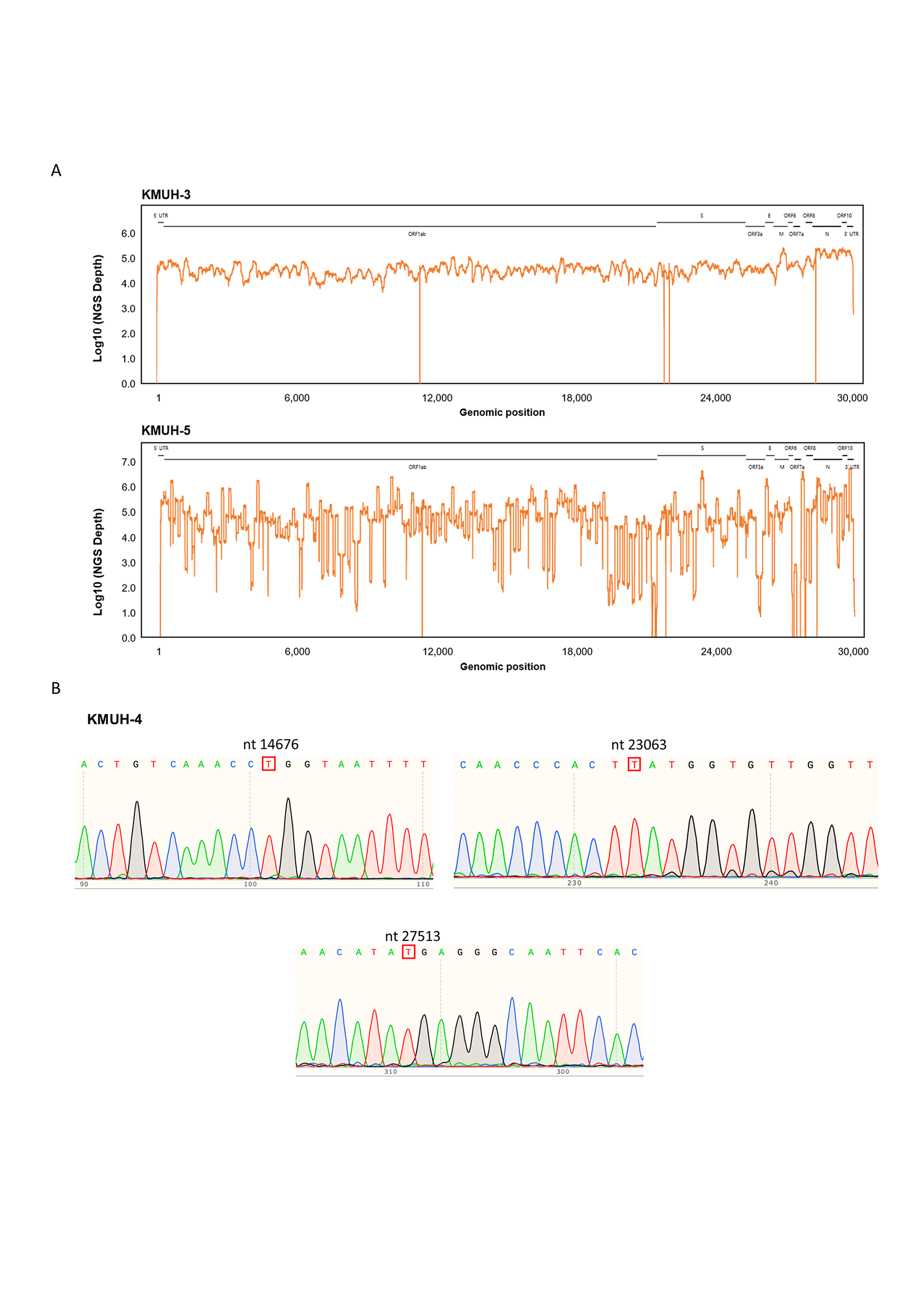

Supplement: Supplementary Figure 2 — A presentation of NGS coverage, depth and SNVs of SARS-CoV-2. (A) NGS coverage and depth of KMUH-3 (RNA-seq) and KMUH-5 (targeted sequencing) according to the genomic positions of the reference strain Wuhan-Hu-1/2019 (MN908947). (B) SNVs at nt positions 14,676 (NSP12 P4804P), 23,063 (spike N501Y), and 27,513 (NS7a Y40Y) were confirmed by Sanger sequencing (KMUH-4). [file Image_2.TIF]

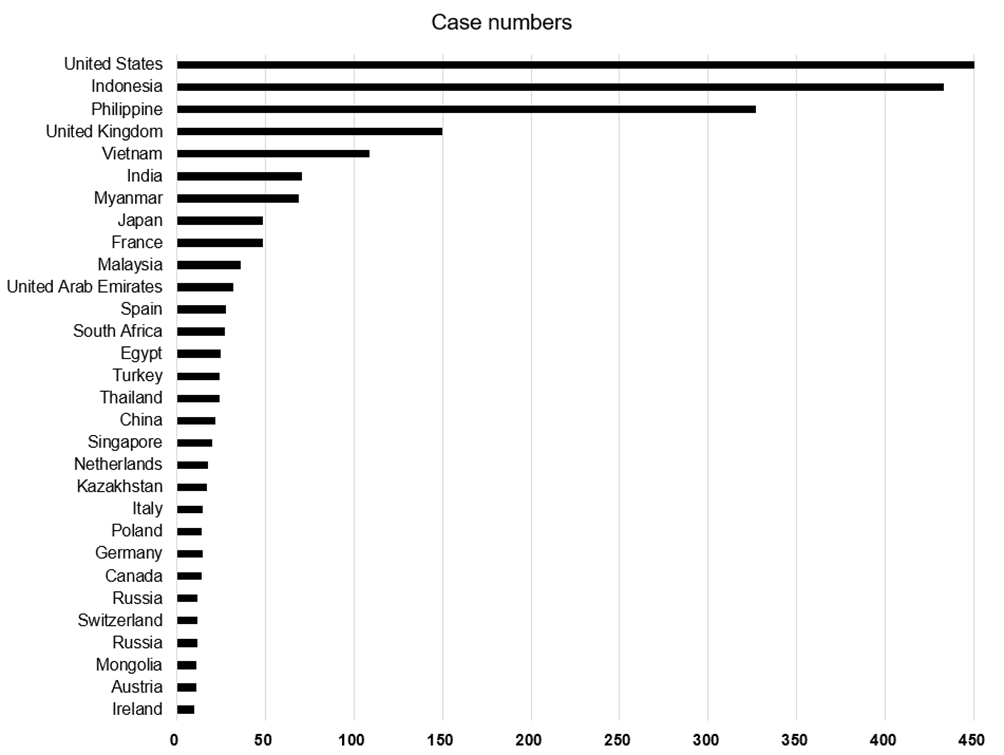

Supplement: Supplementary Figure 3 — Accumulating imported COVID-19 cases from different countries between January 2020 and December 2021 in Taiwan. Source of data: https://nidss.cdc.gov.tw/nndss/disease?id=19CoV. [file Image_3.TIF]
